# Remodeling of Root Growth Under Combined Arsenic and Hypoxia Stress Is Linked to Nutrient Deprivation

**DOI:** 10.3389/fpls.2020.569687

**Published:** 2020-10-23

**Authors:** Vijay Kumar, Lara Vogelsang, Romy R. Schmidt, Shanti S. Sharma, Thorsten Seidel, Karl-Josef Dietz

**Affiliations:** ^1^Department of Biochemistry and Physiology of Plants, Faculty of Biology, University of Bielefeld, Bielefeld, Germany; ^2^Department of Biosciences, Himachal Pradesh University, Shimla, India; ^3^Department of Plant Biotechnology, Faculty of Biology, University of Bielefeld, Bielefeld, Germany; ^4^Department of Botany, School of Life Sciences, Sikkim University, Gangtok, India

**Keywords:** root hairs, meristem, phosphate, iron, hypoxia, arsenic, redox, ROS

## Abstract

Root architecture responds to environmental stress. Stress-induced metabolic and nutritional changes affect the endogenous root development program. Transcriptional and translational changes realize the switch between stem cell proliferation and cell differentiation, lateral root or root hair formation and root functionality for stress acclimation. The current work explores the effects of stress combination of arsenic toxicity (As) and hypoxia (Hpx) on root development in *Arabidopsis thaliana*. As revealed previously, combined As and Hpx treatment leads to severe nutritional disorder evident from deregulation of root transcriptome and plant mineral contents. Both As and Hpx were identified to pose stress-specific constraints on root development that lead to unique root growth phenotype under their combination. Besides inhibition of root apical meristem (RAM) activity under all stresses, As induced lateral root growth while root hair density and lengths were strongly increased by Hpx and HpxAs-treatments.

A dual stimulation of phosphate (Pi)-starvation response was observed for HpxAs-treated plant roots; however, the response under HpxAs aligned more with Hpx than As. Transcriptional evidence along with biochemical data suggests involvement of *PHOSPHATE STARVATION RESPONSE 1*; *PHR1*-dependent systemic signaling. Pi metabolism-related transcripts in close association with cellular iron homeostasis modulate root development under HpxAs. Early redox potential changes in meristematic cells, differential ROS accumulation in root hair zone cell layers and strong deregulation of NADPH oxidases, NADPH-dependent oxidoreductases and peroxidases signify a role of redox and ROS signaling in root architecture remodeling under HpxAs. Differential aquaporin expression suggests transmembrane ROS transport to regulate root hair induction and growth. Reorganization of energy metabolism through NO-dependent alternate oxidase, lactate fermentation, and phosphofructokinase seems crucial under HpxAs. TOR and SnRK-signaling network components were potentially involved in control of sustainable utilization of available energy reserves for root hair growth under combined stress as well as recovery on reaeration. Findings are discussed in context of combined stress-induced signaling in regulation of root development in contrast to As and Hpx alone.

## Introduction

Root development is a dynamic and highly regulated process (Scheres et al., 2002; Hodge et al., 2009; Petricka et al., 2012). The developmental plasticity of root systems allows plants to establish a sustainable organ tailored to the prevailing environmental conditions. The simple root architecture in seedlings changes to an elaborate post-embryonic root development program in maturing plants. However, fine-tuning of the plant root architecture is required upon stress exposure at any life stage. Molecular understanding of root development and underlying gene regulatory networks presently evolves using genomic and molecular biological approaches. Several transcription factors under control of nutrients, redox and reactive oxygen species (ROS) or hormones have been identified (Shin and Schachtman, 2004; Hodge et al., 2009; Ruiz Herrera et al., 2015).

Root growth plasticity is largely determined by temporal fluctuations in the balance between cell division and cell differentiation (Perilli et al., 2012; Sozzani and Iyer-Pascuzzi, 2014). While cell division in the meristems is mostly regulated by auxins, the differentiation-promoting cytokinin (CK) can inhibit cell division through *ARR1* transcription factor (Moubayidin et al., 2010). *ARR1* activates a negative regulator of *PIN* (auxin transport facilitators) i.e., *SHY2*. ROS participate in controlling the process (Tsukagoshi et al., 2010; Yang et al., 2018). Further, modification of stem cell growth through programmed cell death occurs under severe water stress (Cao and Li, 2010).

Environmental stimuli like nutrient deprivation, salt or water stress modulate the growth phenotype of primary roots, lateral roots as well as root hairs (RHs) (Müller and Schmidt, 2004; De Tullio et al., 2010; Liu et al., 2015; Satbhai et al., 2015). RHs are well-studied structures particularly in seedlings as a model for exploring the genetic control of cell division and differentiation, spatiotemporal onset of regulatory mechanisms and epigenetic influence on development processes (Grierson and Schiefelbein, 2002; Grierson et al., 2014). Their structural simplicity makes them ideal for mechanistic understanding of development (Grierson et al., 2014; Salazar-Henao et al., 2016). Different aspects of RH development are thoroughly scrutinized for regulatory mechanisms especially under nutrient deprivation, e.g., epidermal cell differentiation into trichoblast (RH- or H-cell) and non-trichoblast (N-cells), RH initiation and tip growth, cell wall modifications as facilitators of tip growth and ectopic RH growth (Müller and Schmidt, 2004; Shin et al., 2005; Bruex et al., 2012; Kwon et al., 2015; Shibata and Sugimoto, 2019).

Besides widely studied phosphate- (Pi) and Fe-starvation, interference between N- and K-uptake and N-assimilation has also been recognized as important factors in RH development (Müller and Schmidt, 2004; Salazar-Henao and Schmidt, 2016; Huang et al., 2020). Recent studies described functions of ethylene in RH development among other growth regulatory hormones (Katsumi et al., 2000; Cho and Lee, 2013; Neumann, 2016; Huang et al., 2020). Complex gene regulatory networks determine the response to nutrient deprivation especially for Pi and Fe (Cui S. et al., 2018). However, their functional specificity and relative importance in an event of co-occurrence of multiple nutrient deprivations or other environmental factors remain elusive, especially in adult plants.

Identification of mechanisms underlying plant responses to naturally co-occurring multiple stresses requires experiments where plants are exposed to these stress combinations (Suzuki et al., 2014). The responses and mechanisms governing acclimation to combinatorial stresses often differ from regulatory pathways involved in single stress acclimation (Rasmussen et al., 2013; Suzuki et al., 2014). Unique transcriptomic, proteomic, and metabolic changes are evident in plants exposed to combined stresses (Pandey et al., 2015; Suzuki et al., 2016).

Metabolic and molecular responses of Arabidopsis to a naturally prevalent combination of arsenic (As) and hypoxia (Hpx) were recently described by Kumar et al. (2019). Arsenic contamination is a major problem linked worldwide to either groundwater or irrigation of rice cultivated in affected areas (Naujokas et al., 2013). Numerous agricultural fields and groundwater reservoirs in South East Asia, Europe or North America are highly contaminated with As (Medunić et al., 2020). Remediation of contaminated sites using hyperaccumulator plants and generation of As-tolerant but non-accumulating crops, particularly in edible parts, is the need of hour (Kofroňová et al., 2018). However, co-occurring environmental factors aggravate As toxicity, tolerance and accumulation in crop plants and impede growth potential of As-hyperaccumulators. For example, flooding is required during the early stages of rice cultivation.

The use of As-containing groundwater aggravates pollution in the contaminated areas. Indeed, the As-enriched micro-ecosystem represents a complex environment involving several inherently interacting factors, e.g., low soil pH (increased CO_2_), pH-induced As(V) reduction to As(III), and especially Hpx for root growth. Not only Hpx, but subsequent re-oxygenation, when soil dries up, acts as another stress factor. These combined factors likely interfere with As uptake, accumulation including grain deposition and toxicity in rice. A combination of As and Hpx imposes distinct influence on plants due to unique and overlapping features of these stressors. For example, a significant part of the characteristic signaling pattern under As and Hpx is mediated by ROS (Pucciariello et al., 2012; Islam et al., 2015), while both simultaneously affect the cellular energy metabolism (Bailey-Serres and Voesenek, 2008, 2010).

Application of HpxAs stress to *Arabidopsis thaliana* generated unique responses at physiological, transcriptomic, and metabolic level apart from certain overlapping changes (Kumar et al., 2019). Besides characterizing primary stress effects in the roots, it was possible to analyze the dynamics of rapid root-to-shoot communication (Kumar et al., 2019). The most challenging scenario that developed under HpxAs was strong deregulation of nutrient homeostasis. It is known that both stresses affect nutrient uptake (Blokhina and Fagerstedt, 2010; Zhao et al., 2010); however, under HpxAs, plants were challenged to acclimate to As under Hpx-induced inhibition of energy metabolism.

Root growth was severely inhibited during combined stress treatment; however, growth recovery was evident on reaeration (Kumar et al., 2019). In fact, the combined stressed plants showed a lag in root growth recovery compared to individual treatments. Besides a unique and strong downregulation of many transcripts (>500), about 300 other transcripts increased in expression specifically under As and Hpx combination (Kumar et al., 2019). All these observations indicated existence of an energy efficient way to balance root stem cell division and differentiation in a way to best utilize the available resources for coping with nutrient deprivation and to facilitate sequestration of As for stress acclimation.

The present study scrutinizes the root growth phenotype under combinatorial exposure to As and Hpx. Meta-analysis of root growth-related genes specific for combined stress led to the identification of transcriptional adjustments indicative for the remodeling of root architecture to achieve Hpx and As adaptation. Particularly, transcripts involved in root meristem proliferation and cell differentiation displayed pronounced changes. The root phenotype suggested a strong stimulation of epidermal cell differentiation into RHs. Biochemical measurements and transcriptomic data indicate a major role of redox regulation in observed changes in root architecture, while treatment-dependent decrease in epidermal and cortical cell viability was identified as an acclimation tradeoff.

## Materials and Methods

### Plant Growth and Stress Application

*Arabidopsis thaliana* (Col-0; WT) plants were grown hydroponically as described previously (Kumar et al., 2019). In brief, the plants were grown on 0.25 strength Hoagland nutrient medium (1.25 mM KNO_3_, 0.5 mM NH_4_H_2_PO_4_, 0.75 mM MgSO_4_, 1.50 mM Ca(NO_3_)_2_, micronutrients, 14.5 μM Fe-EDTA, 500 μM MES (pH 5.25) for 32 days (10/14 h day/night; 100 μmol photons m^–2^ s^–1^ at 22/19°C (day/night, 50% relative humidity). The nutrient medium was constantly aerated (ambient air 21% O_2_) and renewed weekly. For stress application, plant roots after 32 days culture were exposed to 250 μM As(V; Na_2_HAsO_4_), Hpx or HpxAs. For Hpx, the nutrient medium was pre-flushed with air from a nitrogen generator for 48 h (99.6% N_2_ and 0.4% O_2_) to achieve average O_2_ concentration of 86 ± 4.3 nmol ml^–1^ compared to 262 ± 6.8 nmol ml^–1^ for control and As (mean ± SE, *n* = 15 experiments). This ensured an immediate hypoxic stress. The nitrogen flushing was continued during the treatment period of 7 days, while control plants and those exposed to As alone were aerated as before (21% O_2_). The oxygen content remained stable over the 7 days treatment period. For reaeration, the hypoxic media was replaced with normally aerated one, however presence of As was continued in HpxAs. It is important to add that the shoots remained in air under ambient light conditions throughout. Plant roots (whole) were harvested after respective treatment and re-aeration time points in liquid nitrogen. The stored (−80°C) plant material was used for biochemical assays. Plant shoot and roots were also measured for their fresh weight after 7 d of treatment.

### Microarray and Quantitative Transcript Analysis

The raw microarray data analyzed in this study can be accessed from NCBI GEO^1^ and was published first by Kumar et al. (2019). They detailed the method for RNA extraction, DNA hybridization, qRT-PCR and data analysis. In brief, plant roots (whole) were pulverized in liquid nitrogen and RNA was extracted to be used for microarray-based transcriptome analysis as well as for quantitative analysis of transcript amounts using qRT-PCR. Quantity and quality of the RNA were tested with the NanoDrop ND-1000 spectrophotometer and by gel electrophoresis (Aranda et al., 2012). RNA quality for microarray hybridization (Affymetrix Arabidopsis Gene 1.0 ST arrays) was again tested prior to hybridization using the Agilent 2100 bioanalyzer system. All samples had RNA integrity numbers ≥ 9. Hybridization was done by KFB, Center of Excellence for Fluorescent Bioanalytics (Regensburg, Germany; www.kfb-regensburg.de). The summarized probe set signals in log2 scale were calculated by using the RMA algorithm (Irizarry et al., 2003) with the Affymetrix Gene Chip Expression Console v1.4 Software. The transcriptome data was initially filtered for transcripts belonging to the three major GO-related terms i.e., “Root and Root Hair Development,” “Phosphate Starvation Response,” and “Fe-Homeostasis” and showing linear fold change of −2 ≥ fc ≥ 2 for at least one treatment. The identified transcripts were further sub-categorized into those directly related to RH development, root growth-related hormonal signals, root meristem maintenance, cell wall modification, hypoxia or As-dependent phosphate starvation response, Fe-homeostasis, NO-generation, and ROS producing and scavenging enzymes required for root growth regulation. Further, the transcripts in these sub-categories were filtered for false discovery rate (FDR ≤ 5%) adjusted *p*-value ≤ 0.05 for treatment effect. In the next and last filtering step, those FDR-filtered transcripts which showed a unique expression under HpxAs or had relevance to HpxAs-induced root growth phenotype were converted in suitable heat maps or histograms, while complete lists appear in supplements. Brief description of transcript changes, under As, Hpx and HpxAs-treatments, which relates to cell wall modification and lipid metabolism has been given in supplements by Kumar et al. (2019).

It is pertinent to add that the validity of microarray data has been tested through qRT-PCR for multiple arsenic and hypoxia-stress markers as well as through their recovery after e.g., removal of hypoxia by Kumar et al. (2019). Further, qRT-PCR analysis for additional hypoxia markers and their stress recovery has been done in this study and data presented in Supplementary Figure 2B. For qRT-PCR, RNA samples were processed to generate cDNA. Transcripts were quantified on MyiQ qPCR cycler (BIO-RAD) using KAPA SYBR qPCR master mix and target specific primers (Supplementary Table 5). PCR efficiency and transcript quantification calculation were based on analysis using LinRegPCR 11.0 software (Ramakers et al., 2003; Ruijter et al., 2009). Gene expression was normalized by calculating the normalization factor from the geometric mean of actin (*ACT2*) and tubulin (*TUB5*) expression as described (Vandesompele et al., 2002).

### Root Growth Phenotype and Confocal Imaging

#### Root Hair Phenotype

**FIGURE 1:**
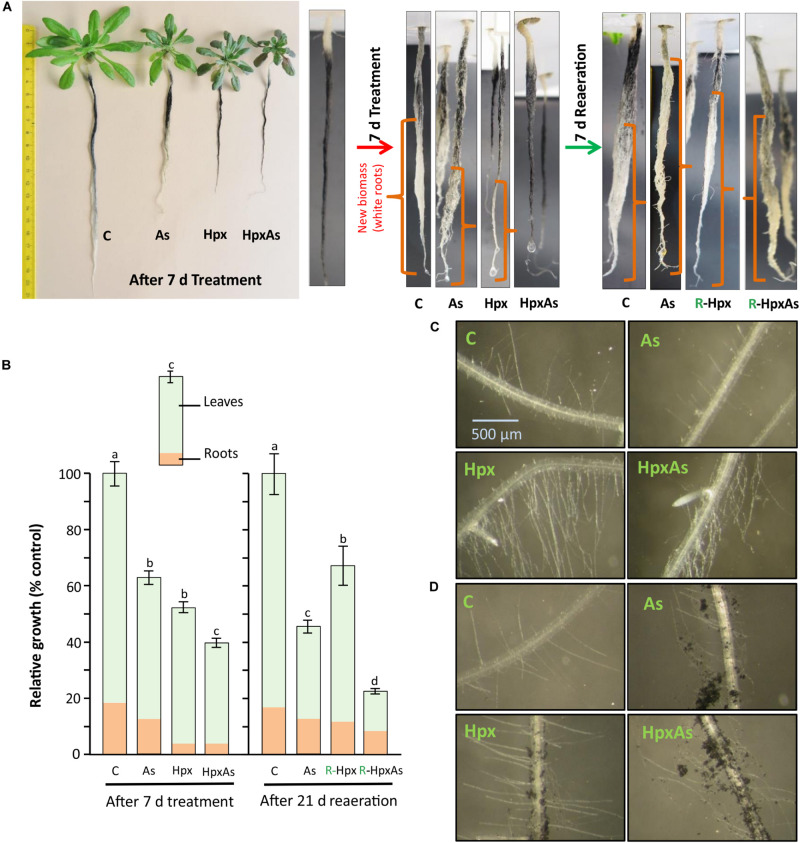
Root and root hair phenotype of plants exposed to arsenic (As), hypoxia (Hpx) and their combination (HpxAs). *A. thaliana* plants were grown for 32 days in hydroculture and then exposed to As (V; 250 μM), Hpx or their combination (HpxAs) for 7 days. **(A)** Plant images (left panel) after 7 days of treatment for comparison of treatment effects on root and shoot growth. Just before the start of treatment and start of reaeration, plant roots were stained with active charcoal. The strong surface adherence of charcoal particles allows assessment of fresh root growth during treatment and reaeration, which appear white. Close-up images of roots (middle) highlight newly formed root biomass after 7 days treatment. In these close-up images roots are not in scale for comparison of total root length however can be compared qualitatively for extent of growth (in different root zones) among different treatments (white color). Also shown are images of stained roots (right) that were reaerated for 7 days after treatment (supplemented with aerated nutrient media; supplemented with As(V) in case of HpxAs-treatment). **(B)** Data on relative total biomass accumulation (shoot and root visible) after 7 days of treatment (means ± SE, *n* = 40; 3 independent experiments; Tukey’s test, *p* < 0.05) and long reaeration (21 days) (means ± SE, *n* = 12; Tukey’s test, *p* < 0.05). Further, images of the RH zone under different treatments without **(C)** or with **(D)** charcoal staining after 7 days treatment.

A quantitative measure of treatment effects on root growth in terms of total accumulated fresh weight is presented in Figures 1A,B. However, to identify the regions of new biomass accumulation (taproot, lateral roots, or root hairs) and comparative extent of growth during the 7 d treatment period, a qualitative measurement of root growth was done for two independent experiments. At the start of treatment period, the plant roots were dipped in well stirred active charcoal suspension for 5 min and rinsed with water (Schat and Ten Bookum, 1992). This charcoal staining allows highly effective analysis of root growth during treatment period without interfering with nutrient uptake (Schat and Ten Bookum, 1992). After 7 d, the growth phenotype was recorded, and the roots were freshly stained with active charcoal before start of reaeration (Figure 1A). Further, we took a closer look at RH growth under a Leica MZ6 modular stereomicroscope with 4x zoom and an artificial light source. RH zone (65–80 areas) from multiple plants per treatment belonging to two independent experiments were photographed and compared for RH length and density. Representative images are presented in Figure 1. The RH images were also recorded after treatment of charcoal-stained roots.

#### Cell-Viability Analysis

Viability of different cell layers in the RH zone was tested with the fluorescent dye SYTOX Green (Truernit and Haseloff, 2008). SYTOX fluorescent dye only penetrates non-viable cells (permeable plasma membranes) wherein interaction with DNA increases their fluorescence >500 fold, reducing the background appreciably (Truernit and Haseloff, 2008). The confocal imaging (LSM 780, Carl Zeiss, Germany) was carried out on the 7th day of treatment. Intact plant roots were immersed with SYTOX green (250 nM dilution from 5 mM stock in DMSO) for 7 min followed by thorough washing with water. SYTOX green in the labeled roots was excited using the argon multiline laser (488 nm, 25 mW) with corresponding main beam splitter and the emission recorded between 500–550 nm (emission max. 523 nm). At 10X magnification (Zeiss Plan-Apochromat 10x/0.45 M27) with a pixel dwell time of 1.2–2.2 μs and a data depth of 12 bits per pixel, 15–20 images were recorded per treatment and experiment. Imaging was carried out within 30 min of staining, when the signal intensity was maximum. Images were evaluated with the ZEN software for fluorescence intensities. The signal-to-noise ratio was optimized for the treatment with maximal fluorescence intensity and the gain (master) and pinhole values were kept constant throughout the treatments. Fluorescence intensity values for the selected excitation channel were collected separately from three different regions of interests (epidermal/cortical cell layers) within the imaged RH zone avoiding saturated areas. The collected data are presented in form of box plots as well as fold differences among treatments.

#### Fluorescence Analysis for Reactive Oxygen Species (ROS)

In plant growth and development, ROS generation drives processes like cell wall synthesis and root tip growth. However, a strict control over the intensity of ROS generation is important to protect cells from their deleterious effects. For *in vivo* ROS staining, 2’, 7’-dichlorodihydrofluorescein diacetate (H_2_DCFDA) was used (Eruslanov and Kusmartsev, 2010). H_2_DCFDA converts to the highly fluorescent 2’, 7’-dichlorofluorescein (DCF) upon cleavage of the acetate groups by esterases and subsequent oxidation. Intact roots after 7 d of treatment were dipped in DCFDA (5 μM from 10 mM stock in DMSO) for 5 min and then washed with water. The argon multiline laser (488 nm, 25 mW) was used for H_2_DCFDA excitation, while emission was collected between 500–550 nm (emission max. 517–527 nm). Representative images are given in Supplementary Figure 4.

### Biochemical Analysis

#### Ferric Chelate Reductase Activity

The activity of ferric chelate reductase was measured in whole roots using a spectrophotometric measurement of purple-colored Fe(II)-ferrozine complex (Emre and Koiwa, 2013). The whole plant roots after 7 d of treatment were dark incubated in Fe(III)-EDTA solution (0.1 mM) containing ferrozine ([3-(2-pyridyl)-5, 6-diphenyl-1, 2, 4-triazine sulfonate]; 0.3 mM) for 45 min. The activity proceeds at room temperature in a 2 ml tube with occasional inversion to assure that root surface stays in touch with solution. After incubation, the roots were rinsed several times in the same solution, and liquid droplets were removed. Absorption of the formed purple complex was quantified at 562 nm against the assay reagent. The calculation of the enzyme activity was based on the molar extinction coefficient of the complex (28.6 mM^–1^ cm^–1^) and pre-recorded fresh weight of roots.

#### Activities of Hypoxia-Responsive Enzymes of Energy Metabolism

Hypoxic growth conditions inhibit normal energy metabolism and induce fermentation. Committed enzymes are pyruvate decarboxylase (PDC), alcohol dehydrogenase (ADH) and lactic dehydrogenase (LDH). PDC activity was measured as conversion of pyruvate to acetaldehyde in presence of its cofactors thiamine pyrophosphate, Mg^2+^ and enzyme ADH (Mithran et al., 2014). The extraction buffer comprised of Na-Pi buffer (50 mM, pH 7.0) supplemented with MgCl_2_ (5 mM), EDTA (5 mM) and thiamine pyrophosphate (TPP, 500 μM). Further, phenylmethylsulphonyl fluoride (PMSF, 100 μM), dithiothreitol (DTT, 1 mM) and leupeptin (5 μM) were added to increase protein stability. Approx. 30 mg of root tissue was used for enzymes extraction with 70 μl of extraction buffer. The extraction was done using Precellys^®^ homogenizer (6,800 rpm, 3 cycles of 20 s with 30 s pause in between). The homogenized material was centrifuged at 15,000 × *g* (4°C) for 15 min. The assay mixture (117 μl) contained 95–100 μl MES (50 mM, pH 6.0), 2 μl NADH (670 μM), 5 μl ADH (1.2 U/ml), 5–10 μl of root extract and 5 μl sodium pyruvate (6 mM). Baseline was adjusted after adding ADH, while addition of pyruvate started the reaction. The activity was measured as decrease in NADH absorption at 340 nm for 3 min. Specific activity was calculated based on molar extinction coefficient of NADH (6.22 mM^–1^ cm^–1^) and protein content in enzyme extracts. Protein content for all the enzyme assays was quantified using Bradford method (Bradford, 1976).

Extraction for ADH and LDH activity tests was performed in 100 mM Tris–HCl (pH 7.7) buffer with EDTA (5 mM), and freshly added DTT (1 mM), cysteine (10 mM), PMSF (100 μM), and 5 μM leupeptin. Enzyme extraction procedure was like PDC except ∼40 mg tissue was extracted with 100 μl buffer. ADH was measured in the direction acetaldehyde to ethanol (Huang et al., 2002). The final assay mixture (115 μl) comprised of 100–107 μl Tris–HCl (100 mM, pH 8.5) with 2 μl NADH (670 μM), 5 μl acetaldehyde and 2.5–10 μl enzyme extract. Baseline was adjusted after addition of acetaldehyde. The reaction was started by addition of enzyme extract and absorption was monitored for 3 min at 340 nm for decrease in NADH amount. LDH activity was measured in the direction pyruvate to lactate (Hanson and Jacobsen, 1984). In addition, 4-bromopyrazole was used to inhibit endogenous ADH. The assay mixture (124.5 μl) comprised 100 mM Tris–HCl (pH 8.0), 2 μl NADH (670 μM), 2.5 μl 4-bromopyrazole (3 M), 10 μl enzyme extract and 10 μl pyruvate (6 mM). The baseline was recorded after addition of extract, while addition of pyruvate started the reaction. Absorbance change of NADH was monitored at 340 nm. NADH molar extinction coefficient was used for calculating specific activity for both ADH and LDH.

#### XTT Reductase Activity

NADPH-oxidases (RBOH; respiratory burst oxidase homolog) form superoxide radicals. Their activity was measured in crude membrane extracts using NADPH-driven and superoxide-dependent reduction of XTT [2,3-bis(2-methoxy-4-nitro-5-sulfophennyl)-2H-tetrazoilum-5-carboxanilide sodium salt] using a modified method (Hao et al., 2006; Potockỳ et al., 2012). The crude extraction of the plasma membrane proteins needed two different buffers i.e., (a) extraction buffer (EB) containing Tris–HCl (50 mM, pH 8.0), sucrose (250 mM), and EDTA (80 mM) and freshly added PMSF (100 μM), and (b) resuspension buffer (RB) comprising MOPS (50 mM, pH 7.2) supplemented with 250 mM sucrose and PMSF (100 μM). In the first step of extraction, ∼30 mg root tissue was added with 70 μl of EB and homogenized in Precellys^®^ homogenizer (2 times 3 cycles of 6,800 rpm, 20 s each with 30 s pause every time). The homogenate was centrifuged at 16,000 × *g*, 15 min (4°C). The supernatant was discarded, and the pellet washed with 1 ml of EB. The tube was inverted to resuspend the soluble proteins. After a second centrifugation (16,000 × *g*, 15 min, 4°C), the pellet was resuspended in 50 μl RB. The vortexed suspension was transferred with a blunt end pipette tip to a 1.5 ml tube and was incubated at 30°C for 15 min. After centrifugation (16,000 × *g*, 5 min, 4°C), the crude PM extract was ready in the supernatant and was used for activity analysis. The assay mixture had 60 μl of Tris–HCl (50 mM, pH 7.5), 10 μl XTT (5 mM in DMSO), 10 μl NADPH (2 mM), and 10 μl of protein. The NADPH specificity of the reaction was tested by excluding NADPH from the reaction which completely stopped XTT reduction. Superoxide dismutase (SOD) served as specific inhibitor of the XTT reduction by quenching superoxide. The activity was recorded for 5 min. The supernatant from the first centrifugation (soluble cell lysate) was also used in order to determine the extent of XTT reduction in soluble cell lysate. The protein content estimation for the crude extract did not work with the Bradford assay, so the activities are presented on fresh weight bases. The calculation of XTT reduction or NADPH oxidase activity were based on ε = 21600 M^–1^ cm^–1^ for XTT at 470 nm (Able et al., 1998).

#### Activities of Antioxidant Enzymes

Enzyme extraction was done in 300 μl of HEPES buffer (100 mM, pH 7.6, freshly added with 100 μM PMSF) for 30–50 mg of root tissue. The homogenization in Precellys^®^ homogenizer was performed 2 times, 3 cycles of 6,800 rpm, 20 s each with a pause of 30 s. The homogenate was centrifuged at 10,000 × *g* for 10 min (4°C). Ascorbate peroxidase (APX) activity was measured immediately after extraction. Assay mixture (115 μl) for APX activity comprised 83 μl HEPES (50 mM, pH 7.6), 7 μl ascorbate (ASC, 5 mM), 10 μl enzyme extract and reaction started by adding 15 μl H_2_O_2_ (400 μM final concentration). Decrease in ASC absorbance was measured for 3 min at 290 nm. Activity calculations were based on ε_ASC_ = 2.8 mM^–1^ cm^–1^. The same enzyme extract was used for guaiacol peroxidase (PER) activity. In this reaction the peroxidase oxidizes guaiacol and activity can be recorded as absorbance change at 470 nm (Amako et al., 1994). In a reaction volume of 223 μl, 200 μl K-Pi buffer (100 mM, pH 6.8), 10 μl guaiacol (0.5% in H_2_O), 3 μl extract and 10 μl H_2_O_2_ (12 mM stock) were added. Addition of H_2_O_2_ started the reaction and the change in absorbance was measured for 5 min. Activity was calculated per unit protein using ε = 22.6 mM^–1^ cm^–1^ for oxidized guaiacol.

Catalase activity was measured in the same extract polarographically using a Clark-type O_2_ electrode (Goldstein, 1968). The reaction mixture contained 890 μl of HEPES buffer (100 mM, pH 7.6) along with 100 μl of H_2_O_2_ (100 mM). The reaction was started by addition of 10 μl of enzyme extract and rate of evolution of oxygen was recorded for 5 min.

#### Plant Cell Sap Osmolarity

For determination of osmolarity of cell sap, tissue sap was squeezed out from fresh root and leaf tissue (50–100 mg) using micro pestle. The extract was centrifuged (16,000 × *g*, 10 min) and supernatant diluted suitably to be measured for osmolarity on automatic semi-micro osmometer (A0800, Knauer). The osmometer was calibrated before measurements with 0 and 400 mosmol/l NaCl solutions.

Residual inorganic phosphate (Pi) after 7 d of treatment was measured in the nutrient media using malachite green-based quantification (Baykov et al., 1988). The reagents used were (a) malachite green (1 mM) with polyvinyl alcohol (0.16%) in H_2_SO_4_ (6 mM) and (b) ammonium molybdate (50 mM) in H_2_SO_4_ (3.4 M). The reagents were mixed freshly before measurement in a ratio of 1:0.5 (a: b). The measurements were carried out in a plate reader with 198 μl reagent mix and 2 μl sample. Absorbance was measured at 620 nm after thoroughly mixing the contents in each well. In parallel the assay was also calibrated with a Pi-concentration series (0–5 μM; NH_4_H_2_PO_4_).

### Statistical Analysis

Data are presented as means with standard error. Statistical analysis (one-way analysis of variance; ANOVA followed by Tukey’s *post hoc* test) was performed to evaluate significant differences among means using IBM SPSS Statistics for Windows, version 20 (IBM Corp., Armonk, NY, United States). Significantly different means (*p* < 0.05) are marked with different letters while missing letters indicate lack of significance. The statistical analysis for the microarray data was performed as part of complete transcriptome analysis by Transcriptome Analysis Console version 3.1 (Affymetrix).

## Results

Several studies report that As stress alters the plant redox state and nutrient availability (Carbonell-Barrachina et al., 1997; Srivastava et al., 2005; Zhao et al., 2010; Duan et al., 2013). Similarly, it is known that Hpx along with generating challenges for energy metabolism and redox homeostasis also alters nutrient uptake (Dat et al., 2004; Bailey-Serres and Voesenek, 2008; Blokhina and Fagerstedt, 2010). Further, HpxAs combined stress induced a profound deregulation of nutrient homeostasis (Kumar et al., 2019). The results presented here identify nutrient deregulation-associated signaling patterns in relation to root growth.

### Root Growth

Arabidopsis plants grown hydroponically for 32 days were exposed to As, Hpx and their combination (HpxAs) for 7 days. It has been shown that total root biomass accumulation was affected by these treatments (Figures 1A,B). Especially for Hpx and HpxAs, biomass accumulation was significantly reduced (Figures 1A,B). To estimate the extent and pattern of new root growth during the treatment period, plant roots were also stained with active charcoal. The images presented in Figure 1A reveal the differential pattern of root growth among different treatments. Compared to control plants, main root growth was inhibited to a certain extent in As and lateral roots were generated. Root growth inhibition seemed complete under both Hpx and HpxAs. Plant roots under Hpx showed a slight enlargement of the tap root tip, although growth was severely inhibited. No such root tip growth was visible for plants under HpxAs. Charcoal staining was also carried out after 7 d treatment before start of reaeration and plant roots were again photographed after one week of reaeration in order to judge the severity of applied stresses and plant potential for recovery from Hpx. It became clear from these observations that the root growth inhibition could recover after reoxygenation of the nutrient media (Figure 1A). However, total biomass accumulation and growth recovery as recorded after 3 weeks of reaeration were slower for HpxAs than in the single Hpx treatment (Figure 1B).

Roots were investigated for RH growth qualitatively (Figures 1C,D). RHs increase root surface area and play important roles in increasing nutrient uptake and maintaining osmotic balance. Comparative imaging of the RH zones revealed a strong stimulation of RH growth in Hpx- and HpxAs-treated plants. Although, it was clear from previous data related to root fresh weight and charcoal staining that root growth showed close to complete inhibition under Hpx and HpxAs, it is revealed here that RH growth was not only sustained, but stimulated compared to As and control. This pattern of root growth inhibition and RH stimulation was also evident in charcoal-stained roots (Figure 1D). Control plants emerged a certain number of RHs in the newly developed RH zone, while the RH zone of treated roots displayed adsorbed charcoal as an evidence of reduced root growth. Also, a higher density and elongation of RHs in Hpx and HpxAs was observed. The stimulated RH growth could be an adaptive strategy to keep up nutrient uptake to sustain plant growth under these stresses.

### Transcriptional Regulation of Root Architecture

**FIGURE 2:**
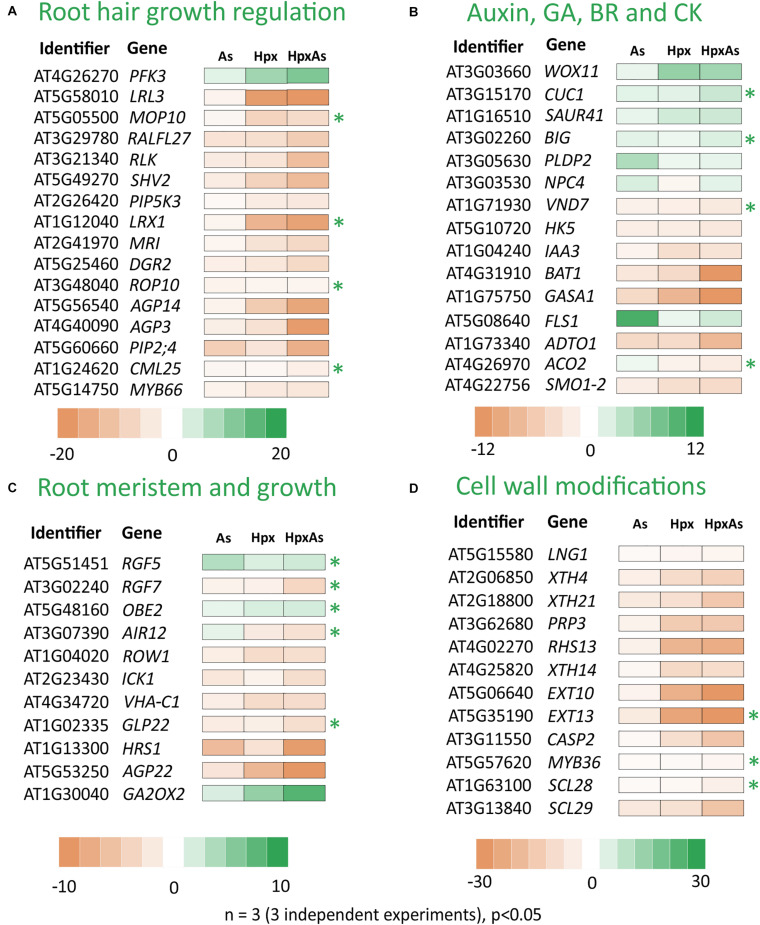
As-, Hpx-, and HpxAs-dependent changes in transcripts linked to root growth and related signaling. Heat maps present root transcriptome data from HpxAs-treatment experiments (GEO-NCBI accession number GSE119327) (Kumar et al., 2019). The transcripts were compared for changed expression under As, Hpx and HpxAs treatment. Four functional categories of genes are presented namely those related to **(A)** RH growth regulation and epidermal cell differentiation, **(B)** biosynthesis and signaling of hormones involved directly in different aspects of root development especially linked to auxins, gibberellic acid (GA), brassinosteroids (BR) and cytokinin (CK), **(C)** control of root meristem activity and total root growth, and **(D)** cell wall growth or modification crucial in RH or lateral root development. The transcripts were initially selected with the GO term “Root development” using the criterion –2 ≥ fc_linear_ ≥ 2 and later sub-categorized. Among transcripts with a significant response (FDR *p* ≤ 0.05) to the treatments, heat maps present those with unique response to HpxAs or those with relevance to observed HpxAs-stress effects on root development. Transcripts with unique response to HpxAs (–2 ≥ fc_linear_ ≥ 2 for HpxAs, FDR *p* ≤ 0.05 and either response below this threshold for As, Hpx or response statistically not-significant) are marked with an asterisk. Supplementary Table 1 gives the complete gene list with relative expression under different treatments.

To identify mechanistic players possibly involved in the altered root phenotype, root transcriptome data were queried for genes related to root development and their stress responses. A total of 281 genes were filtered for a linear fold change of −2 ≥ fc_linear_ ≥ 2 (Supplementary Table 1). The identified genes were grouped into four major categories as shown in Figures 2A–D. Among the identified transcripts, categories “root hair growth regulation” “hormonal signaling,” and “root meristem and growth maintenance” represented roughly 30% each of total identified genes, while the rest of the around 10% belonged to “cell wall modifications.” Sets of 11 to 16 transcripts were selected and presented in four heat maps (Figure 2) based on further filtering with FDR *p* ≤ 0.05 for the expression change and their response or relevance to HpxAs-treatment.

HpxAs-treated plants showed a strong inhibition of root growth with a simultaneous stimulation of RH density and tip growth. It is significant that besides 86 genes, which were categorized to be causally related to RH growth, several transcripts in other categories were also involved with RH signaling, epidermal cell differentiation or RH tip growth via rapid cell wall formation. A sizable proportion of the differentially regulated root development-related transcripts was associated with RH differentiation, RH growth and its regulation. Significantly, of the 220 genes from above showing a change of −2 ≥ fc_linear_ ≥ 2 for HpxAs, the majority (180) consisted of those with decreased expression. Complete lists of all these selected genes are given in Supplementary Table 1.

Many transcripts in the “root hair growth regulation” category (Figure 2A) with decreased expression importantly function in negative regulation of RH elongation like plasma membrane intrinsic protein 2;4 (*PIP2;4*) and calmodulin like 25 (*CML25). PIP2;4* which encodes an H_2_O_2_-conducting transmembrane aquaporin (Dynowski et al., 2008) decreased 15.2-times for HpxAs compared to control, a response significantly different from As (−9.17), and Hpx (not significant) treatments. Similarly, expression of *CML25* decreased 2.92-times for HpxAs, a unique response compared to other treatments, where no change in expression was recorded. The Rac-like GTP-binding protein ROP10 is active in cell wall hardening on the sides of elongating RH that in turn generates a force to propel the growing RH tip (Hirano et al., 2018). Its transcript amount decreased 2.02-times under HpxAs, while no response was observed for As and Hpx. ROP10 facilitates phosphatidylinositol-4-phosphate 5-kinase 3, PIP5K3 function in RH growth (Hirano et al., 2018). Importantly, 6-phosphofructokinase 3 (*PFK3*), involved in epidermal cell fate determination i.e., H- and N-cell differentiation, increased strongly for HpxAs (12.39-fold) and Hpx (9.15-fold); magnitude of increase being lower for As (2.99) (Figure 2A). In addition, the root and hypocotyl epidermal cell fate determining protein *WERWOLF1* or *MYB66*, another negative regulator of RH cell growth (Wang et al., 2019), showed stronger decrease for Hpx (−4.1) and HpxAs (−4.55) compared to As (−2.26).

Slightly different response patterns arose for hormonal signaling-related transcripts compared to the other three categories (Figure 2B). More than 35% of the total 64 genes that responded to HpxAs were increased in expression; however, still higher number of genes showed reduced expression. Among those with increased expression, *WUSCHEL* related homeobox 11 or *WOX11* and *CUP-SHAPED COTYLEDON1* or *CUC1* are important in auxin-mediated plasticity of root architecture and lateral root formation, respectively (Baesso et al., 2018). *WOX11* expression increased strongly under both Hpx (6.2-fold) and HpxAs (5.46-fold) while no change was detected for As. On the other hand, *CUC1* showed HpxAs-specific increase (3.18-fold), while no change in expression was observed for As and Hpx. No change in expression was observed for two functionally antagonistic genes namely, phospholipase D P2 (*PLDP2*) and the phosphoesterase *NPC4* under Hpx and HpxAs while both increased in expression by 4.67- and 2.33-fold, respectively under As-treatment. Both genes are targeted by auxin signaling and regulate RH density and elongation under nutrient deprivation, especially phosphate (Su et al., 2018).

Among the genes in category “root meristem and growth,” the highlight of the HpxAs-specific response are three root growth factors, i.e., *RGF5*, *RGF7*, and *OBERON* 2 (*OBE2)*, involved in maintenance of root stem cell niche and root meristem identity (Figure 2C). *RGF5* increased significantly under HpxAs (2.35-fold), while *RGF7* decreased in expression by 3.8-times. The change in expression was either not significant or no change was observed for As and Hpx. Further, *OBE2* increased specifically in HpxAs (2.11-fold), while no change in expression was observed for As and Hpx-treatments. Further, auxin-induced gene in root cultures *i.e.*, *AIR12*, another gene involved in lateral root morphogenesis, decreased specifically under HpxAs (−2.82). The negative regulator of root meristem cell number *GA2OX2* (gibberellin 2-beta-dioxigenase) transcript increased strongly under Hpx (5.23-fold) and HpxAs (8.26-fold), potentially limiting root meristem cell number (Figure 2C; Li et al., 2017). Another crucial gene in this category *i.e.*, *REPRESSOR OF WUSCHEL1* (*ROW1*), regulates the identity of quiescent center cell layers by limiting expression of the *WUSCHEL*-related gene *WOX5* (Zhang et al., 2015). Quite interestingly, its expression was reduced 3.39- and 3.01-times under Hpx and HpxAs-treatments, respectively (Figure 2C). This might explain, in a large part, the suppression of root growth observed due to these treatments (Figures 1A,B).

A major portion of cell wall modification-related transcripts showed a strong decrease in expression especially under HpxAs (Figure 2D). For example, several RH cell specific extensin family members like *EXT10* and *EXT13* as well as the casparian strip membrane domain protein like *CASP2* showed strongly decreased transcript amounts under HpxAs which was 75. 25-, 61-, and 16.63-times, respectively (Figure 2D). The expression of these genes was also lower under Hpx and As, however, the extent of decrease was smaller. Expression of the transcription factor *MYB36* which regulates transcription of CASPs in control of differentiation of endodermis, was specifically reduced under HpxAs (−2.65). *SCL28*, encoding a scarecrow-like protein potentially involved in radial root growth regulation, also showed HpxAs-specific 4.25-times decrease in expression. Several other cell wall biogenesis-related genes showed stronger response to HpxAs (Figure 2D).

### Phosphate Starvation Response

**FIGURE 3:**
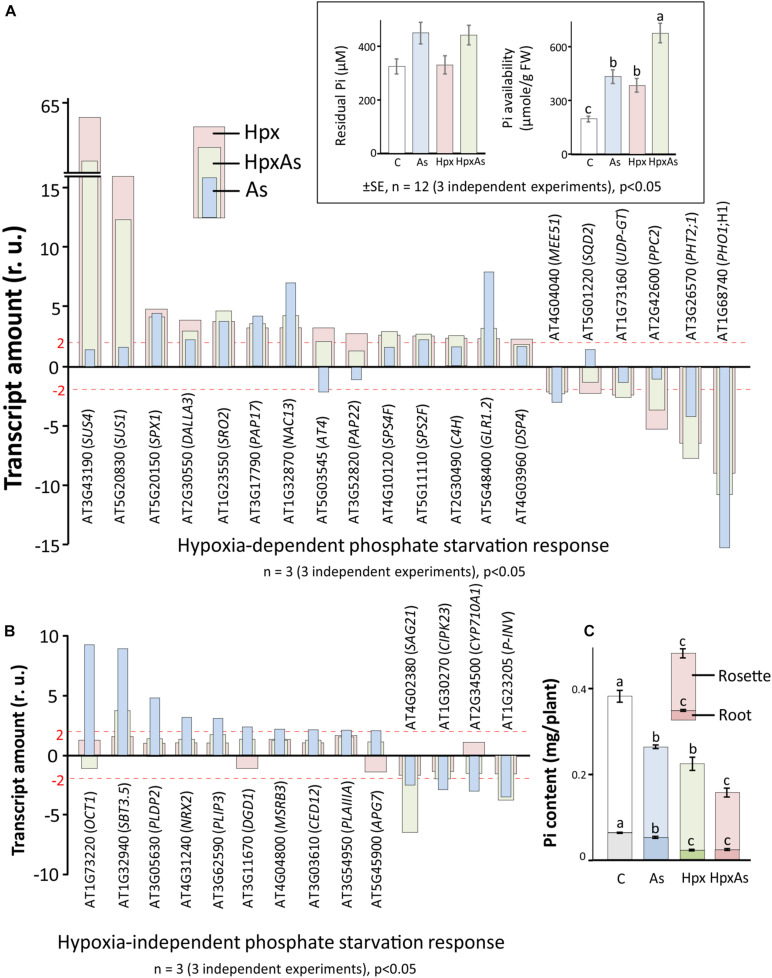
As-, Hpx-, and HpxAs-induced phosphate starvation response in roots. Phosphate starvation-related transcripts were first screened for significant changes (–2 ≥ fc_linear_ ≥ 2, FDR *p* ≤ 0.05) and subsequently for specificity of their response to hypoxia. Phosphate starvation response comprises genes not only related to uptake, transport, and assimilation of inorganic phosphate, but also those involved in galactolipid and sulfolipid biosynthesis, as well as phospholipid metabolism. The genes were categorized into those that showed **(A)** significant changes under Hpx-exposure and **(B)** no-response to Hpx, but significant response to As. The inset depicts the residual Pi in the nutrient media after 7 days of treatment given along with calculated Pi-availability per gram tissue based on the total biomass accumulated per pot at the end of treatment. The complete list with relative expression values for phosphate starvation-related genes are given in Supplementary Table 2. In addition, heat map and a complete list of additional transcripts related to lipid signaling and phosphatidylinositol metabolism which are not part of Figures 2, 3 are given in Supplementary Figure 1 and Supplementary Table 3. **(C)** Phosphate content measured using LC-MS/MS (Kumar et al., 2019) is presented. Data is calculated per plant using accumulated dry weight under different treatments (respective Pi-content in root and rosette is also visible) (means ± SE, *n* = 4; 4 independent experiments; Tukey’s test, *p* < 0.05).

In plants, As(V) interferes with phosphate uptake, assimilation, transport and diverse phosphate-dependent processes like protein activation/deactivation by phosphorylation, membrane properties through phospholipids, and ATP-synthesis (Catarecha et al., 2007; Abercrombie et al., 2008). Similarly, hypoxia interferes with phosphate starvation response through deregulation of related transcripts that in turn affects the root growth regulation (Klecker et al., 2014; Neumann, 2016). Due to this overlap, plants facing HpxAs stress should be severely challenged in maintaining phosphate homeostasis. Phosphate starvation-related transcripts were assessed in HpxAs-treated plants (Figures 3A,B) and compared to Hpx- and As-responses. The analysis mainly included the differentially regulated transcripts related to phosphate homeostasis (uptake, transport, and assimilation), galactolipid (glycolipids) and sulfolipid biosynthesis, phosphatidylinositol metabolism and signaling, and sucrose metabolism. A subset of transcripts related to lipid and phosphatidylinositol metabolism is appropriately summarized in Supplementary Figure 1 and Supplementary Table 3. Likewise, the phosphate deficiency-regulated genes with direct involvement in root and RH growth are depicted in Figures 2A–D and Supplementary Table 1 and certain others related to Fe metabolism appear in a subsequent section (Figure 5B, Supplementary Table 4).

The dual stimulation of phosphate starvation response under HpxAs likely necessitates the adoption of an appropriate energy-efficient state to maintain phosphate homeostasis and enable stress acclimation. Thus, the expression of filtered (relevant) transcripts under HpxAs was compared to that induced by As and Hpx alone. Both Hpx and As induced a phosphate starvation response, with a reasonable overlap between As- and Hpx-induced gene expression. Based on their response, transcripts could be divided into two categories: 20 transcripts were Hpx-responsive (Figure 3A), whereas 14 others were unresponsive to Hpx, but responded to As (Figure 3B). In general, the HpxAs-response exhibited a strong overlap and resembled more with Hpx than with As. For example, among 20 genes depicted in Figure 3A, 15 showed a similar increase or decrease for Hpx and HpxAs, while for 14 genes presented in Figure 3B, only 4 showed similarity of expression for As and HpxAs. Among these 4 transcripts, levels of *SBT3*.*5*, coding for the subtilase 3.5 protein, increased 3.8-fold under HpxAs which was much lower than that for As (8.82). Also, in case of *SAG21* transcripts, encoding an MAPK cascade-associated protein, HpxAs-induced reduction in expression (−6.49-times) was stronger than that for As (−2.42). Among the 20 genes undergoing change of expression under Hpx, 10 genes also responded to As (Figure 3A). In summary, it is significant that 15 out of 35 As- and Hpx-induced phosphate starvation-related transcripts did not change under HpxAs compared to control. Key genes among these were phospholipase (*DALLA3*), sulfoquinovosyl transferase (*SQD2*), phosphoenolpyruvate carboxylase (*PPC2*), and digalactosyldiacylglycerol synthase (*DGD1*).

Pi-starvation response is determined by external Pi-availability and internal plant Pi-status. Residual plant Pi-content in the hydroculture nutrient media was measured spectrophotometrically. This was apparently similar for all treatments. However, calculated per unit plant fresh weight, availability of Pi increased substantially under all stresses, most strongly for HpxAs, due to growth inhibition (inset, Figure 3A). On the other hand, estimated internal Pi-content showed a sharp decline for different treatments and was significantly lower for HpxAs leaves than all other treatments (Figure 3C). Pi-contents also lowered in roots for HpxAs but were only significantly different from control and As-treatments, while they were similar for Hpx and HpxAs-treatment (Figure 3C).

### Cell Viability Analysis in the Root Hair Zone

**FIGURE 4:**
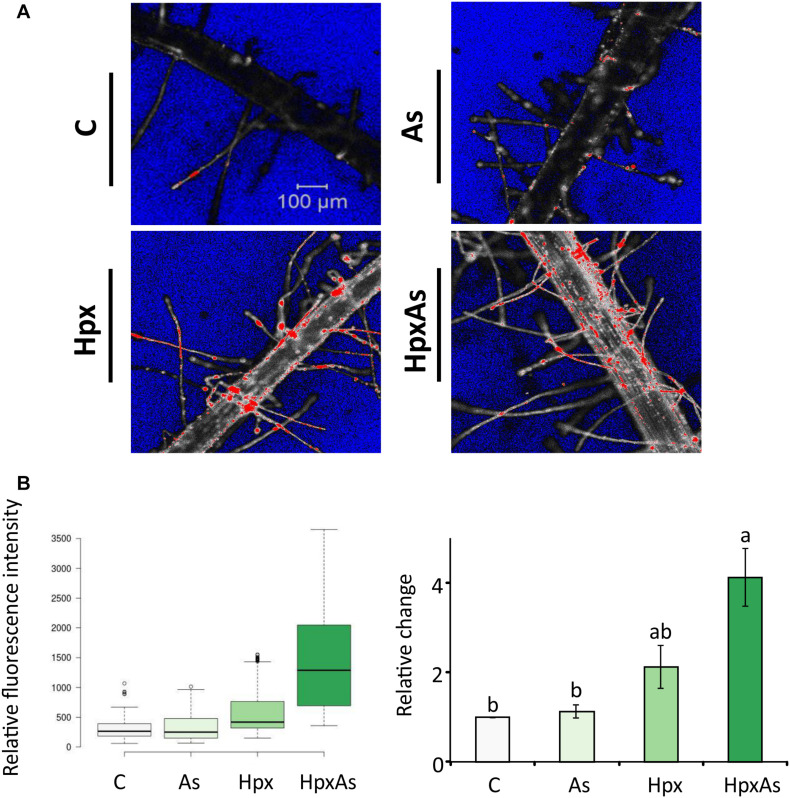
*In vivo* fluorescence analysis for cell non-viability in the root hair zone of As-, Hpx-, and HpxAs-treated Arabidopsis roots. SYTOX green fluorescent probe was used to label non-viable root cells in the RH zone. The roots were exposed to different stresses for 7 days, labeled with SYTOX for 7 min on the last day of treatment and imaged under the confocal laser scanning microscope (LSM780, Zeiss). Fluorescent dye was excited using an argon laser (488 nm) and emission recorded in the range of 500–550 nm. **(A)** The micrographs display the fluorescence of SYTOX green (emission maximum at 523 nm) representing non-viable cells in the RH zone among different applied stresses after 7 days of treatment. The micrographs present fluorescence data in grayscale with added range indicators (Blue; background, Red; fluorescence oversaturated regions) **(B)** Recorded fluorescence intensities were compared for the stresses and are presented as box plot (shiny.chemgrid.org/boxplotr) (left). Center lines show the medians; box limits indicate the 25^th^ and 75^th^ percentiles as determined by R software; whiskers extend 1.5-times the interquartile range from the 25^th^ and 75^th^ percentiles; not connected data points represent outliers. Histogram on the right shows relative changes in SYTOX fluorescence intensity for the treatments compared to control. The data are means ± SE, *n* = 3 (data collected from 3 independent experiments over 250–260 individual RH areas from multiple plants; Tukey’s test, *p* < 0.05). For quantitative fluorescence analysis, saturated areas of the micrographs were excluded.

Root hair imaging and transcriptomic analysis revealed enhanced RH growth to be an important response to HpxAs involving multiple signaling pathways. RH phenotype is determined by multiple crucial steps including epidermal cell differentiation, RH initiation and unidirectional tip growth. Cell viability analysis reveals the physiological status of RHs and underlying epidermal and cortical cells. This could be performed by employing fluorescent probes in a sensitive and non-invasive manner with minimum inadvertent cell damage. The contrast in fluorescence intensity shows a strong loss of cell viability in RH zone (mostly epidermal and cortical cells) of plants treated with Hpx and HpxAs (Figure 4A). A quantitative analysis of several recorded images of fluorescently labeled roots indicated a stronger fluorescence intensity in HpxAs compared to Hpx (Figure 4B). Normalized to the control, we observed a 4.14-fold higher loss of viability in HpxAs plants compared to 2.13- and 1.13-fold for Hpx and As, respectively (Figure 4C). However, due to variation, the difference between Hpx and HpxAs was insignificant.

### Iron Assimilation (and NO Signaling)

**FIGURE 5:**
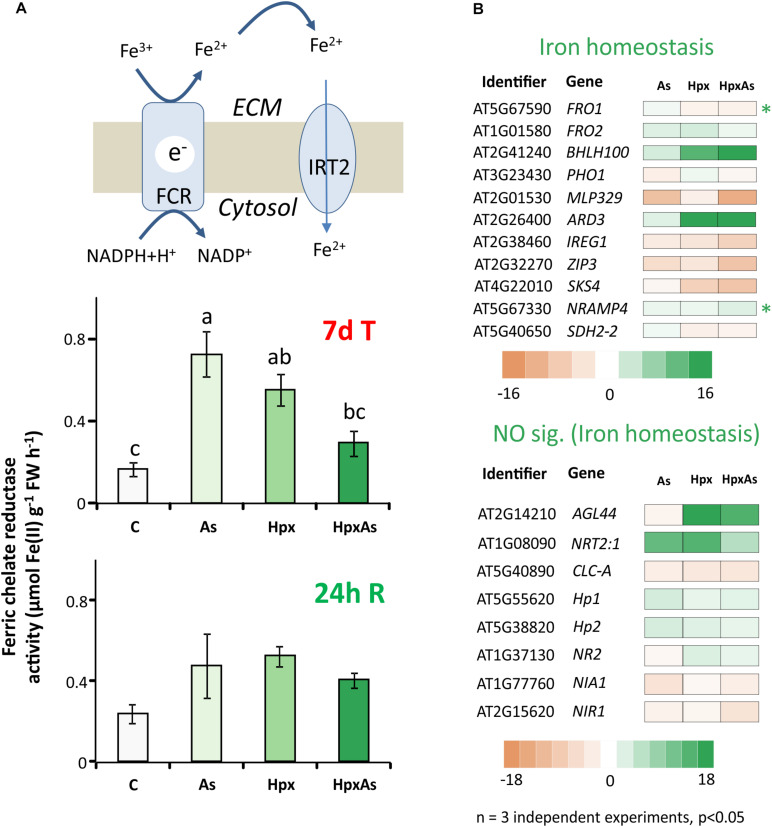
Perturbation of Fe-uptake and assimilation as evident from transcriptomic and biochemical analyses. **(A)** Ferric chelate reductase activity was measured in intact Arabidopsis roots using ferrozine based complexation of Fe(II). The obtained data were compared among different treatments after 7 days of treatment and after additional 24 h of reaeration. The data are means ± SE, *n* = 5, (5 individual plants, 2 independent experiments, Tukey’s test, *p* < 0.05). **(B)** Stress-induced significant changes (–2 ≥ fc_linear_ ≥ 2, FDR *p* ≤ 0.05) in Fe-homeostasis-related transcript amounts are presented in heat maps. The heat maps are categorized based on core Fe- uptake-, assimilation- and metabolism-related genes and those involved in NO generation. They all have a putative role in Fe-homeostasis and root development regulation. Transcripts with unique response to HpxAs (–2 ≥ fc_linear_ ≥ 2 for HpxAs, FDR *p* ≤ 0.05 and either response below this threshold for As, Hpx or response statistically not-significant) are marked with an asterisk. The complete gene lists with transcript amounts under different treatments for Fe and NO are given in Supplementary Table 4.

Stimulatory influence of Pi- and Fe-starvation on RH growth is well studied in the context of regulatory networks. Plants possess two main Fe-acquisition strategies. The first employs the release of specific chelators into the rhizosphere whereas the second uses membrane-spanning enzymes for reduction of rhizospheric Fe^3+^ to Fe^2+^ that is subsequently taken up through Fe(II)-high affinity transporters. Ferric chelate reductases (FCR) are sensitive to changes in cellular Fe-demand and rhizospheric Fe availability. FCR activity was measured spectrophotometrically in intact plant roots to reveal the treatment-specific differences. After 7 d of treatment, As induced a 4.35-fold increase in FCR activity compared to 3.31- and 1.76-fold increase for Hpx and HpxAs, respectively (Figure 5A). The As-induced increase in FCR subsided within 24 h of replenishing the nutrient media (Figure 5A).

To further investigate the perturbation of Fe metabolism in response to As and Hpx, applied individually and in combination, transcriptomes were filtered for transcripts related to Fe-homeostasis. Two heat maps in Figure 5B only show transcript expression for genes regulating Fe-homeostasis in relation to root development and their relevance to HpxAs (for complete list see Supplementary Table 4). Among the 77 identified transcripts (67 related to Fe-homeostasis, 10 to NO generation), 56 responded to HpxAs, where about 50% showed increased expression. Like FCR activity, *FERRIC REDUCTION OXIDASE2; FRO2* increased in expression 2.71-fold under As-treatment, while the response was insignificant under the other two conditions (Figure 5B). Another evidence for induction of Fe-uptake mechanism under As came from a 2.05-fold increase in *IRT2* responsible for Fe(II) uptake downstream to FCR, while no significant change was observed for other treatments. Interestingly, *FRO1*, coding for another FCR gene, decreased specifically under HpxAs (−2). *BHLH100*, another key regulator involved in post-translational regulation of FIT (master regulator of Fe-homeostasis) under JA-signaling (Cui Y. et al., 2018), increased 20.6-fold in HpxAs. *NRAMP4*, involved in Fe-mobilization, increased specifically under HpxAs (2.67-fold) (Figure 5B). Several treatment-responsive transcripts were related to heme binding, nitrate assimilation, and NO-generation (Figure 5B, Supplementary Table 4). NO interferes with Fe-homeostasis and root development (García et al., 2011). Most notable is the Hpx- (3.1-fold) and HpxAs- (2.05-fold) induced increase in the expression of *NR2*, which codes for nitrate reductase and functions as NO synthase.

### Arsenic Interference With Hypoxia Response in Root Stress Adaptation

**FIGURE 6:**
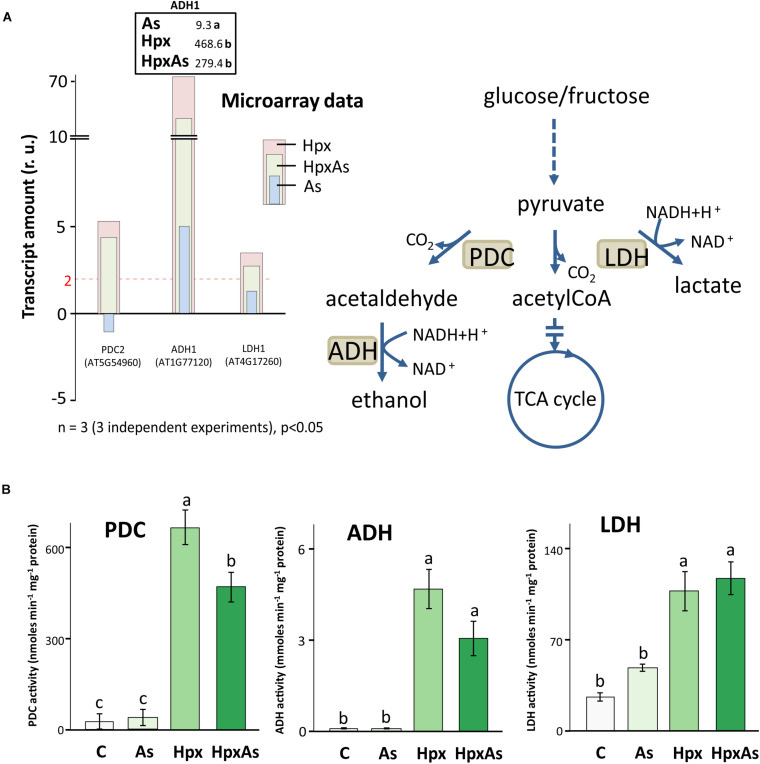
Changes in gene expression and enzyme activity of energy metabolism-related enzymes under HpxAs compared to As and Hpx. Pyruvate decarboxylase (PDC), alcohol dehydrogenase (ADH) and lactate dehydrogenase (LDH) are crucial enzymes under anoxic conditions in ATP generation and regeneration of NAD^+^ when the respiratory electron transport is inhibited. **(A)** The histogram presents relative transcript amounts of *PDC2*, *ADH1*, and *LDH1* genes after 7 days of stress application as compared to control. qRT-PCR confirmation of this transcript accumulation under given stresses is given for *ADH1* on top of *ADH1* bar as fold change numbers, while for several other related Hpx marker genes data are presented in Supplementary Figure 2. Primer sequences for the qRT-PCR data are given in Supplementary Table 5. **(B)** Enzyme activities for PDC, ADH, and LDH were measured spectrophotometrically in root extracts. Data display specific activity as means ± SE, *n* = 6 (3 independent experiments, Tukey’s test, *p* < 0.05).

Under hypoxia, plants carry out lactate and ethanol fermentation to generate low amounts of ATP, but more crucially to regenerate NAD^+^. Thus, plant adaptation and growth under hypoxia rely on these subsidiary energy pathways. Enzymes involved in fermentation are transcriptionally upregulated under hypoxia (Kosmacz et al., 2015). It was reported that presence of As affects the hypoxia-induced increase in transcription of fermentation-related genes like pyruvate decarboxylase (PDC) and alcohol dehydrogenase (ADH) under HpxAs (Kumar et al., 2019). Therefore, the changes in *PDC2*, *ADH1* and *LDH1* were compared with the activities of PDC, ADH and lactate dehydrogenase (LDH). Increase in *PDC2*, *ADH1* and *LDH1* expression was smaller under HpxAs as compared to Hpx-treatment (Figure 6A). *ADH1* also increased by As alone. Microarray data and their qRT-PCR confirmation are given for several other typical Hpx-response markers (Figure 6A, Supplementary Figures 2A,B). Further, the effects of As in HpxAs, on the recovery of these hypoxia markers was also evaluated after reaeration (Supplementary Figure 2B).

Enzyme activity measurements proved the interference of As with Hpx responses (Figure 6B). For example, increase in PDC activity was significantly lower under HpxAs as compared to that under Hpx alone. It increased 24- and 16.9-fold under Hpx and HpxAs, respectively. A similar trend was observed for ADH, with a 75.0- and 49.2-fold increase in activity under Hpx and HpxAs-treatment, respectively; however, they were not statistically different. Arsenic did not alter the activity of both enzymes (Figure 6B). In contrast to PDC and ADH, LDH activity increased similarly in response to HpxAs (4.38-fold) and Hpx (4-fold). Arsenic also increased LDH activity (82%) but the increase was not statistically significant (Figure 6B). The enzyme activity data indicate a preferred upregulation of lactic acid fermentation pathways under HpxAs.

### Redox Regulation of Root Growth

**FIGURE 7:**
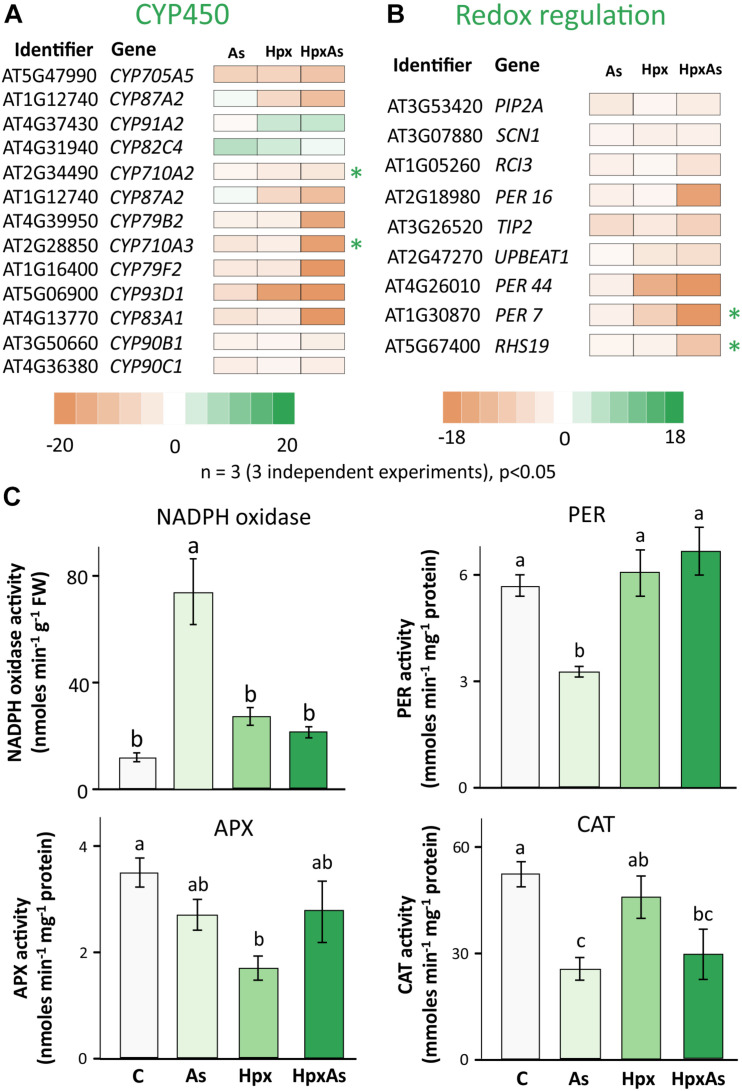
Biochemical and transcriptomic analysis of redox signaling-related regulators of root and root hair growth. **(A)** Cytochrome P450s (CYPs) gene family comprises heme-containing proteins involved in diverse cell metabolic processes, including redox regulation of growth and development. Transcript accumulation as influenced by As, Hpx, and HpxAs is presented in the heat map for different cytochrome P450s. The genes were initially identified in the search for GO-related terms “Root development” and “Fe-homeostasis.” Included transcripts showed a significant change in expression (–2 ≥ fc_linear_ ≥ 2, FDR *p* ≤ 0.05) and are assumed to be relevant for the HpxAs-induced root growth phenotype. **(B)** Redox regulatory transcripts involved in root development, other than CYP450s presented above, were selected for their significant response (–2 ≥ fc_linear_ ≥ 2, FDR *p* ≤ 0.05) and are presented in heat map. Transcripts with unique response to HpxAs (–2 ≥ fc_linear_ ≥ 2 for HpxAs, FDR *p* ≤ 0.05 and either response below this threshold for As, Hpx or response statistically not-significant) are marked with an asterisk. The complete lists for all selected CYP450s and other redox regulatory genes are provided in Supplementary Table 6. **(C)** NADPH dependent XTT reductase activity was measured in crude membrane extracts to analyze stress effects on NADPH oxidases. The extract was also tested for its superoxide specificity by using SOD randomly for certain samples to demonstrate inhibition of XTT reduction (data not shown). Guaiacol peroxidase (PER), ascorbate peroxidase (APX), and catalase (CAT) were measured for their enzyme activity in root extracts. All enzyme activity data are means ± SE, *n* = 6 (3 independent experiments). Micrographs showing ROS generation in different cell layers in the RH zone, based on 2’, 7’-dichlorodihydrofluorescein diacetate (H_2_DCFDA) fluorescence are given in Supplementary Figure 4.

ROS and the associated redox regulatory network are crucial in the control of root meristem activity and growth of roots and RHs (Shin et al., 2005; De Tullio et al., 2010). Several transcripts were identified in the GO-related terms “Root and Root Hair Development” and “Fe-homeostasis” and were further classified into members of cytochrome P450 family (CYP450s) and peroxidases (Figures 7A,B, Supplementary Table 6). A large set of these gene products were extracellular and function in redox regulation of cell wall synthesis, hormonal signaling, and diverse stress responses. Among the identified 109 CYP450 family transcripts, 61 responded significantly to HpxAs, with 85% showing a down-regulation (Figure 7A, Supplementary Table 6). On the other hand, among 30 identified redox regulatory genes, 25 showed significant response under HpxAs-stress and all were down-regulated (Figure 7A, Supplemenatry Table 6).

Many members of the CYP450 family have NADPH-dependent functions and participate in synthesis of brassinosteroid(s) (BR), glucosinolates, camalexin and sterols. C-22 sterols play a crucial role in cell membrane polarity and hence RH initiation and tip growth axis determination (Ovečka et al., 2010). For example, *CYP710A2* and *A3* decreased strongly under HpxAs; while the reduction for *CYP710A2* was 4.65-times, *CYP710A3* decreased 18.4-times (Figure 7A). No significant change was observed under Hpx or As alone. Further, the amount of *CYP83A1*, involved in auxin homeostasis, was also reduced (−81.3) under HpxAs. The same decreased under As (−5.13) and Hpx (−3.35), however, with a significantly lower magnitude. Among other redox regulatory genes, *UPBEAT1 or UPB1*, another bHLH transcription factor, was reduced in expression for Hpx and HpxAs-exposure by 4.16- and 5.55-times, respectively. It is of significance that *UPB1* plays a crucial role in root meristem development through regulation of ROS accumulation in between the zones of cell proliferation and elongation, thus could impede the onset of differentiation on reduced expression (Tsukagoshi et al., 2010; Li et al., 2019).

The root hair cell-specific peroxidases *PER7* and *PER73* (*RHS19*) showed strongly decreased expression (24.8- and 9.98-times, respectively) under HpxAs (Figure 7A). Expression of both did not change significantly under Hpx and As treatments. ROS accumulated in epidermal and cortical cells of the RH zone under Hpx and, more strongly, HpxAs (Supplementary Figure 4). The peroxidase *PER16* was less expressed in HpxAs (−16.32), where the extent of reduction was several folds higher than that under As (−2.45). Transcript amounts of the central regulator of RH initiation SCN1 decreased under Hpx (−2.5) and HpxAs (−2.39), with no change for As (Figure 7A). SCN1 functions through regulation of trichoblast-specific NADPH oxidase *(RBOH-C*) (Arenas-Alfonseca et al., 2018).

Therefore, NADPH-oxidase activity was measured in crude root membrane extracts (Figure 7C). For comparison, XTT-reductase activity was also measured in the soluble supernatant (Supplementary Figure 3) and showed the robust nature of NADPH oxidase activity data as the pattern is quite different in two measurements. More importantly no significant treatment-specific difference was observed for the soluble fraction. NADPH oxidase activity tended to be higher for Hpx (2.30-fold) and HpxAs (1.81-fold) but was not statistically significant (Figure 7C). In contrast, the increase in activity was substantial (6.26-fold) for As-treatment (Figure 7C). Further, activities of different peroxidases showed variable treatment-specific patterns of change, indicating their specific role(s) under different stress regimes. APX activity dropped significantly (50%) under Hpx but decreased only marginally under As and HpxAs (Figure 7C). In case of guaiacol peroxidases, activity decreased (−42%) due to As, and increased marginally due to Hpx (7%) and HpxAs (17%). Catalase activity was significantly lower (51%) only under As treatment (Figure 7C). Treatment and compartment specificity of function for these enzymes is evident from the above observations.

## Discussion

### Suppression of Root Growth With Concomitant Stimulation of Root Hair Development

Stress exposure of hydroponically grown Arabidopsis plants allowed distinguishing root-specific responses to HpxAs-stress combination vis-à-vis the individual treatments. Root exposure to HpxAs induced nearly complete inhibition of root growth that recovered upon reaeration (Figures 1A,B; Kumar et al., 2019). Lateral root suppression under oxygen depletion has been reported recently (Shukla et al., 2019; Pedersen et al., 2020). Root growth suppression due to HpxAs and Hpx typically coincided with a strong stimulation of RH growth (Figures 1C,D). Induction of RH growth serves as an effective strategy to salvage deregulated nutrient uptake by increasing the total root surface area and in turn absorption capability (Gahoonia and Nielsen, 1998; Müller and Schmidt, 2004; Cui S. et al., 2018). A perturbed cellular osmotic balance may also be one of the reasons for RH growth induction (Supplementary Figure 5).

Arsenic interferes with phosphate-uptake and phosphate-dependent cellular processes (Zhao et al., 2010), and also with homeostasis of both macro- (S) and micronutrients (Fe, Zn or Mn) (Rai et al., 2011; Duan et al., 2013; Lee et al., 2013). Hypoxia similarly deregulates nutrient assimilation (Dat et al., 2004; Blokhina and Fagerstedt, 2010). Thus, it is plausible that the transient inhibition of root growth and induction of RH differentiation in response to HpxAs reflect the operation of a regulatory mechanism to sustain nutrient uptake at minimum energy cost and prevent excess As-uptake.

**FIGURE 8:**
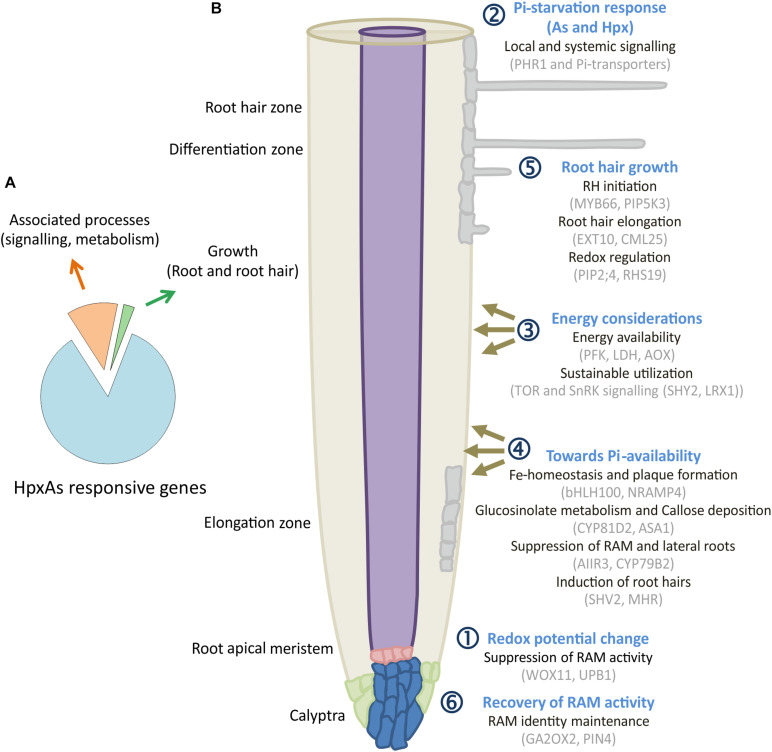
HpxAs-specific changes in root development pattern. **(A)** Pie chart gives distribution of genes identified for their involvement in root and RH development among all the genes with unique transcript deregulation under HpxAs. **(B)** The schematic shows a spatiotemporal arrangement of contemplated events in roots under HpxAs exposure. Events are categorized into six groups with emphasis on role of phosphate homeostasis deregulation and associated redox, low energy state-dependent signaling events. Further studies should be focused on the specific analysis of these described transcripts in order to decipher their particular role in HpxAs-stress acclimation as well as to resolve the temporal information that could lead to better mechanistic understanding. See text for detailed discussion of the processes depicted in this figure.

Of the HpxAs-specific genes, about 20% were associated with root and RH growth regulation (Figure 8A; Kumar et al., 2019). After FDR-*p* value adjustment, additional genes were identified to show unique response to HpxAs, which belonged to the regulatory network of root growth (Figures 2, 5, 7). Interestingly, the transcriptional response to HpxAs was aligned more with that to Hpx rather than As. In general, the fine-tuning of stress acclimation under HpxAs involved a higher proportion of deregulated genes with reduced expression. Delayed root growth recovery from HpxAs upon reaeration may indicate the effect of this expressional deregulation.

### Interaction of Nutrient Homeostasis Networks

#### Phosphate Starvation

Phosphate starvation triggers adjustment of RAM activity and enhanced formation of lateral roots and RHs (Abel, 2011). Thibaud et al. (2010) proposed that external Pi controls 70% of the genes with function particularly in the local response, while internal Pi contents modulate the systemic Pi-starvation response. HpxAs is challenging since As inhibits high affinity phosphate transporters and hypoxia affects shoot Pi-content and/or metabolism (Zhao et al., 2009; Klecker et al., 2014; Kumar et al., 2019). As the external Pi availability was apparently not constrained for HpxAs-treated plants, Hpx-induced phosphate starvation response seems to be regulated systemically. As such, the Pi-starvation response seems to influence internal Pi-redistribution majorly, more than Pi-uptake with significantly different Pi-content for HpxAs in leaves compared to other treatments (Figure 3C). The interpretation is supported by the observation that most of the Pi starvation-related, Hpx-deregulated transcripts function in systemic signaling (Thibaud et al., 2010). Klecker et al. (2014) identified a similar response in hypoxic shoots of seedlings that was regulated by *PHR1* in a light-dependent manner.

Phosphate starvation induces membrane lipid reorganization to replace a major portion of phospholipids with phosphate-free galacto- and sulfolipids (Cruz-Ramírez et al., 2006; Nakamura et al., 2009). A lipid profile of Arabidopsis leaves and roots under HpxAs could shed more light on the consequences of this transcriptional change. Observed phenotypic differences, i.e., As-induced lateral root growth and Hpx-induced RH development might potentially be governed by the contrasting intensity of Pi-starvation response (Figure 1). Additional contribution of other regulatory pathways might be necessary. The gene network downstream to *PHR1* affects iron uptake and accumulation, sulfate fluxes, anthocyanin synthesis, photosynthesis, sucrose synthases, cell division, and plant growth (Steyn et al., 2002; Rouached et al., 2011; Nilsson et al., 2012; Müller et al., 2015). Perturbation of S-fluxes might directly affect the As sequestration, thereby, increasing its toxicity (Seth et al., 2012), while changes in Fe-homeostasis could influence root meristem activity and root architecture (Müller et al., 2015).

#### Fe-Homeostasis and Interaction With NO

Müller et al. (2015) described callose formation and iron accumulation in primary roots as a mechanism to block cell communication among stem cells by inhibition of root growth and stimulation of lateral roots as well as RHs. Such a response is regulated by Pi-starvation (Abel, 2011; Müller et al., 2015). Under phosphate starvation plants also tend to accumulate iron plaques on the root surface in order to concentrate nutrients in adsorbed form (Tripathi et al., 2014). This is evident from the brownish color of roots under As and HpxAs (Figure 1A; Park et al., 2016; Awasthi et al., 2017). As(V) shows high adsorption efficiency on iron plaques (Lee et al., 2013). ROS facilitate Fe(II) oxidation to Fe(III), and this process is stimulated both under Pi-starvation and in presence of As(V) (Liu et al., 2004; Awasthi et al., 2017), resulting in accumulation of iron oxide on root surface. The rapid oxidation of Fe(II), could lead to enhanced FCR activity under As-exposure to restore the Fe(II) availability (Figure 5A).

The situation in HpxAs plants is more complex. A strong and selective deregulation of genes involved in callose deposition, indole glucosinolate biosynthesis and catabolism indicates phosphate starvation-induced callose and iron deposition under different treatments (Figure 5, Supplementary Tables 4, 7) (Müller et al., 2015; Bell, 2018). This might result in the induced lateral growth phenotype in As. Further, selective increase of transcripts involved in glucosinolate biosynthesis and callose deposition (e.g., *CYP91A2*; Hpx, HpxAs, *AGL44*; Hpx, HpxAs, *CYP81D8*; HpxAs, and *ASA1*; HpxAs) coupled with a parallel decrease in abundance of transcripts associated with auxin signaling, root system development, and lateral growth (e.g., *AIR3*; Hpx, HpxAs and *CYP79B2*; HpxAs) might largely explain the observed RH elongation under Hpx and HpxAs (Figures 2, 5, Supplementary Table 7) (Francisco et al., 2016).

In addition, a strong reduction in expression of glucosinolate catabolic enzymes i.e., *TTG4*, *TTG5* (Hpx, HpxAs) and *NSP1*, *NSP4* (all) would cause a glucosinolate build-up (Supplementary Table 7). Glucosinolates function in root growth regulation through facilitation of cell wall callose deposition and hormonal metabolism (Francisco et al., 2016; Bell, 2018). Contrasting FCR expression and activity response in HpxAs plants compared to As-alone could be explained by strongly increased expression of *bHLH100* transcription factor, a negative regulator of Fe-uptake under Fe-starvation (Cui Y. et al., 2018). The strong deregulation of Fe-starvation transcripts seems to have internal homeostatic and redistribution function, as no bifurcation of RH tips was observed under HpxAs, which is otherwise a distinctive Fe-starvation response (Figures 1, 4, Supplementary Figure 4) (Müller and Schmidt, 2004).

### Redox Regulation of Root and Root Hair Growth Under HpxAs

Redox cues are central regulatory components in shaping the root system development (De Tullio et al., 2010; Tsukagoshi, 2016; Mhamdi and Van Breusegem, 2018). HpxAs exposure induced a significant redox potential shift toward oxidized cytosol in the quiescent center and surrounding cell layers within the first 4 h of the start of treatment (Kumar et al., 2019). The shift under HpxAs differed from that in As and Hpx and diverged further during the 7 d treatment (Kumar et al., 2019). This redox potential shift might constitute the first trigger for the changing root development program. After 7 d of treatment, expression of several genes involved in stem cell niche maintenance, control of stem cell proliferation and differentiation and RH initiation and growth were specifically modulated under HpxAs (Figures 2, 7).

Three components of the redox regulatory network seem crucial for root development and stress acclimation under HpxAs namely, (a) activation of NADPH oxidases and ROS generation, (b) maintenance of balance between ROS generation and quenching and (c) downstream perturbations by ROS accumulation and redox potential changes (e.g., altered heme and Fe-S cluster synthesis, deregulation of secondary metabolic enzymes of family CYP450s, hormone metabolism). Among the NADPH oxidases (Mittler et al., 2004), *RBOHA* and *RBOHD* increased in all treatments and *RBOHB* only in Hpx (Kumar et al., 2019). *FRO4*, another NADPH oxidase-like transcript, decreased under all treatments. Effects on *FRO4* were stronger in HpxAs than in Hpx and As (Kumar et al., 2019). Measured NADPH oxidase activity, in total root membrane extract, increased significantly due to As (Figure 7C). Involvement of NADPH oxidases in As-induced oxidative damage and growth reduction has been reported (Gupta et al., 2013). The increased NADPH oxidase activity might be responsible for induction of lateral root growth under As-treatment through RAM inhibition (Orman-Ligeza et al., 2016).

NADPH oxidase activity under Hpx and HpxAs was only marginally higher than that in the control. Under these stress conditions, NADPH oxidase may play different roles due to perceived changes in oxygen amounts (Lalucque and Silar, 2003; Schmidt et al., 2018). NADPH oxidases has been implicated in ROS accumulation and redox signaling under hypoxia (Wang W. et al., 2018). ROS accumulation was higher in the epidermis and cortex than in the endodermis and pericycle of RH zone under Hpx and HpxAs in contrast to As and control (Supplementary Figure 4). NADPH oxidases play important functions in RH growth and in RAM activity (Kim et al., 2019). Analyses of plants expressing redox sensors like roGFP2 in conjunction with NADPH oxidase inhibitors might provide insight into the NADPH involvement in RH development under HpxAs (Foreman et al., 2003; Gutscher et al., 2008; Lukyanov and Belousov, 2014).

Besides membrane-associated NADPH oxidases, several other heme-based enzymes like peroxidases and oxidoreductase of the CYP450 family function in generation, interconversion, and quenching of ROS. The HpxAs-specific downregulation of many of the CYP450 transcripts and peroxidases could be a part of cellular redox fine tuning. Measurement of the APX, guaiacol PER, and CAT activities in total soluble root protein extracts suggested them to be differentially involved in response to the applied stressor(s) (Figure 7C). HpxAs imposed a substantial decline in cell viability in the cortical and epidermal cell layers in the RH zone. Stress-dependent ROS accumulation could lead to lipid peroxidation and damage to plasma membrane (Farmer and Mueller, 2013). However, considering the specific roles of ROS in facilitating tip growth of RHs (Mangano et al., 2017; Wang S.S. et al., 2018), the observed loss of cell viability could also be induced by programmed cell death (PCD), thus having a regulatory relevance (Petrov et al., 2015).

Lower transcript amounts of *MARIS* under Hpx and HpxAs could explain the viability differences, at least for the epidermal cells, as MARIS is a RH membrane protein and positive regulator of membrane integrity (Boisson-Dernier et al., 2015). It is intriguing that *UBP1*, which regulates peroxidase activity and ROS accumulation in the zone between cell proliferation and cell elongation, decreased in expression. The gene product is a negative regulator of cell division (Li et al., 2019), however, its involvement in the development of HpxAs-root phenotype is not clear. Analysis of expression kinetics and protein activity change could be expected to provide important clues. The altered glutathione availability and redox potential (Kumar et al., 2019) might perturb heme and Fe-S cluster synthesis (Hider and Kong, 2013). Downstream changes in activity of heme-containing proteins would considerably impact the redox signaling cascades (Ryter and Tyrrell, 2000; Hider and Kong, 2013).

### Transcripts Associated With Remodeling of Root Architecture Under HpxAs

A significant number of deregulated transcripts were related to root growth inhibition, stem cell maintenance for recovery (Hpx and HpxAs), lateral root growth/inhibition (As/HpxAs, respectively), stimulation of RH density and elongation (Hpx and HpxAs). Figure 8B depicts a spatiotemporal scheme of envisaged events occurring in roots under combined stress exposure leading to observed remodeling of root architecture. The figures also compile some of the transcripts associated with the six consecutive steps. Subgroups in each category are provided with exemplary sets of proteins which are expected to control root development under HpxAs and during subsequent reaeration. Early redox changes served as primary stress sensory event leading to initial suppression of root apical meristem (RAM) proliferation.

Subsequent dual induction of Pi-starvation response in stressed Arabidopsis further suppressed RAM activity during 7 d treatment. Further, energy considerations led plants to suppression of lateral root growth but induced root hair growth for sustainable nutrient uptake. Redox- and ROS-dependent regulation seems to act primarily in root hair growth and could also be responsible for loss of epidermal and cortical cell viability. Due to increased transcription of genes coding for meristematic cell maintenance-related proteins, plants were able to recover primary root growth to a certain extent. However, a strong downregulation of transcription for a larger set of genes seems to result in lag in recovery for HpxAs-treated plants compared to Hpx (Figure 1B). Three categories of transcripts appear crucial for signaling and plant adaptation to HpxAs.

#### Stem Cell Management

The majority of these genes participate in auxin signaling, and others carry out cytokinin- (CK) and gibberellic acid- (GA) mediated function. Wuschel-related homeobox genes *WOX11* and *WOX5*, work in successive steps of root founder cell establishment and root primordium development in adventitious rooting (Hu and Xu, 2016). However, the synchronous increase of *WOX11* and decrease of gene expression for *WOX5* regulatory protein *ROW1* under Hpx and HpxAs might be important in maintaining developmental plasticity. Similarly, another stem cell proliferation and differentiation regulator *UBP1* revealed reduced transcription which will favor proliferation along with overlapping increase (*SAUR41*, *GA2OX2*) and decrease (*PIN4*, *VHA-c1*) of other transcripts involved in stem cell maintenance and root development. The result of these changes i.e., increased abundance of root hairs despite root growth inhibition and subsequent growth recovery with a lag under HpxAs could be a fine balance of their activity. In addition, reduced expression of root meristem growth factor *RGF7* under HpxAs might be a crucial factor in growth arrest. Finally, reduced expression of mitogen activated protein kinase kinase *MKK6* under Hpx and HpxAs could participate in inhibition of lateral root growth (Zeng et al., 2011).

#### Energy Constraints and Stress Acclimation

Energy constraint is an important issue dealt by the HpxAs-treated plants, where plant metabolism and growth need to optimally adapt to low oxygen availability along with investment in As detoxification measures. A strong overlap in transcriptomic response to Hpx and HpxAs is consistent with the same. Although further research is needed to elucidate the specific mechanism(s) of stress acclimation, following three observations related to energy metabolism are intriguing.

(a) Increased expression of *NR2* (Hpx, HpxAs) under hypoxic conditions has been linked to AOX activity that drives mitochondrial ATP generation (Vishwakarma et al., 2018). AOX has a distinct role of stimulating the hemoglobin (Hb)-NO cycle to improve energy status under hypoxic conditions. It needs to be added here that *Hb1* gene expression was significantly lower under HpxAs than Hpx, leading possibly to a bigger energy deficit (Supplementary Figure 2).
(b) The phosphofructokinase (PFK)-catalyzed reaction is a tightly regulated committing step of glycolysis. In hypoxic conditions, PPi-dependent PFK is preferred over ATP-dependent PFKs (Bailey-Serres and Voesenek, 2008). Here, transcripts for the ATP-dependent PFK3 increased strongly under Hpx (9.15) and HpxAs (12.39); and its physiological significance needs to be understood (Figure 2A, Supplementary Table 1). It is known that expression of *PFK3* along other kinases is stimulated by histone deacetylase (HDA18) and that they are involved in epidermal cell fate determination (Liu et al., 2013). Enzyme assays suggest lactic acid fermentation to be a preferred fermentation pathway under HpxAs, while the activity of PDC and most likely that of ADH was lower under HpxAs than Hpx alone (Figure 6; Bailey-Serres and Voesenek, 2008).
(c) TOR and SnRK form a central hub in intracellular and extracellular nutrient and energy sensing involved in optimal resource utilization and sustainable growth (Robaglia et al., 2012; Jamsheer et al., 2019). Observed deregulation under HpxAs concerned the following factors with diverse functions: SHY2 is a negative regulator of root growth linking auxin, CK and BR regulation of root meristem. LRX1 interferes with cell wall and RH morphogenesis and is regulated by MAML-4. MLO15 participates in cargo delivery to the plasma membrane during tip growth. RSL4 controls several RH genes. DGR2 interferes with ABA-ROP10 signaling and root morphogenesis. CAP1 controls Ca^+^ gradients for RH tip growth (Weiste et al., 2017; Schoenaers et al., 2018; Schaufelberger et al., 2019; Van Leene et al., 2019; Li et al., 2020; Zhu et al., 2020). All these signaling components downstream of TOR and SnRK signaling may control RH growth under Hpx and HpxAs.

#### Epidermal Cell Differentiation and Root Hair Development

Besides transcripts described above, several RH morphogenesis (*SHV2*, *MHR1*, *2*, *3*, *6*) and RH-specific (*RHS3*, *10*, *12*, *15*, *16*) genes were deregulated under Hpx and HpxAs, in contrast to As, indicating an active and precise control of RH morphogenesis. Their gene products regulate different aspects of RH tip growth like cellular streaming, plasma membrane composition or cell wall growth. For example, transcripts of negative regulator of RH elongation, *PIP2;4*, decreased under HpxAs. PIP2;4 is proposed to function as H_2_O_2_-conducting aquaporin and may considerably contribute to the observed phenotype (Figures 1, 4 and Supplementary Figure 4) (Dynowski et al., 2008). Also, higher RH density phenotype in HpxAs and Hpx may depend on decreased expression of *MYB66* or *WERWOLF1*, a master regulator in determination of epidermal cell fate (Song et al., 2011; Wang et al., 2019).

## Conclusion

The present study observed a nutrient-deprivation induced and redox-regulated root architecture remodeling under HpxAs. Multiple transcripts involved in root development were identified to be specifically deregulated under HpxAs and potentially responsible for observed root hair growth phenotype. Observed Pi-starvation response and downstream changes in Fe-homeostasis for HpxAs-treated plants highlight an intriguing overlap between As and Hpx. Accumulation of ROS in different cell layers, biochemical analyses and cell viability measurements in RH zone indicated a crucial role of redox regulation in root development under HpxAs. Further research should focus on correlation between redox transients, stem cell activity and cell differentiation. Interestingly in HpxAs-treated plants, a significant part of the deregulated root hair growth-related transcriptome belonged to TOR and SnRK-signaling network. Further analysis for functional specificity of these transcripts under HpxAs could reveal regulatory pathway involved in sustaining growth and acclimation response.

## Data Availability Statement

The datasets analyzed for this study can be found in the NCBI-GEO (https://www.ncbi.nlm.nih.gov/geo/query/acc.cgi?acc=GSE119327) (Kumar et al., 2019).

## Author Contributions

VK, TS, SSS, and K-JD planned the study. VK, LV, and TS carried out the experimental work. VK, TS, SSS, K-JD, and RRS interpreted data and discussed the results. VK and K-JD wrote the manuscript and all authors proofread the manuscript.

## Conflict of Interest

The authors declare that the research was conducted in the absence of any commercial or financial relationships that could be construed as a potential conflict of interest.

## Footnotes

1 https://www.ncbi.nlm.nih.gov/geo/query/acc.cgi?acc=GSE119327

## Supplementary Material

Supplementary Figure 1 Heat map for comparison of stress-induced changes in transcript amounts for genes associated with lipid biosynthesis and signaling as well as phosphatidylinositol metabolism.

Supplementary Figure 2 Quantitative RT-PCR analysis results for transcript amounts of hypoxia markers.

Supplementary Figure 3 XTT reductase activity in soluble protein extract.

Supplementary Figure 4 Images from DCFDA fluorescence-based analysis of ROS generation in Arabidopsis roots under As, Hpx and HpxAs.

Supplementary Figure 5 Plant cell sap osmolarity in differentially treated plant roots and leaves.

Supplementary Table 1 Lists of all transcripts related to root and root growth regulation and signaling as influenced by As, Hpx and HpxAs.

Supplementary Table 2 Deregulation of phosphate starvation-related transcripts under applied stresses.

Supplementary Table 3 Lipid biosynthesis and signaling- and phosphatidylinositol metabolism-related transcripts with stress-induced changes in transcript amounts.

Supplementary Table 4 Lists of genes related to Fe-homeostasis and NO formation in regulation of Fe-homeostasis and stress effects.

Supplementary Table 5 Lists of primers used for qRT-PCR quantification of hypoxia marker genes.

Supplementary Table 6 As-, Hpx- and HpxAs-induced significant transcriptional deregulation for cytochrome P450 genes associated with redox regulation of root growth.

Supplementary Table 7 Transcript deregulation for genes related to glucosinolate metabolism.
