# Supplementary material for: Remodeling of Root Growth Under Combined Arsenic and Hypoxia Stress Is Linked to Nutrient Deprivation
Source: Front Plant Sci. 2020 Oct 23;11:569687. doi: 10.3389/fpls.2020.569687 (PMC7644957; doi:10.3389/fpls.2020.569687)
Supplement: Supplementary Figure 1 — Heat map for comparison of stress-induced changes in transcript amounts for genes associated with lipid biosynthesis and signaling as well as phosphatidylinositol metabolism. [file Presentation_1.PPTX]

## Slide 1
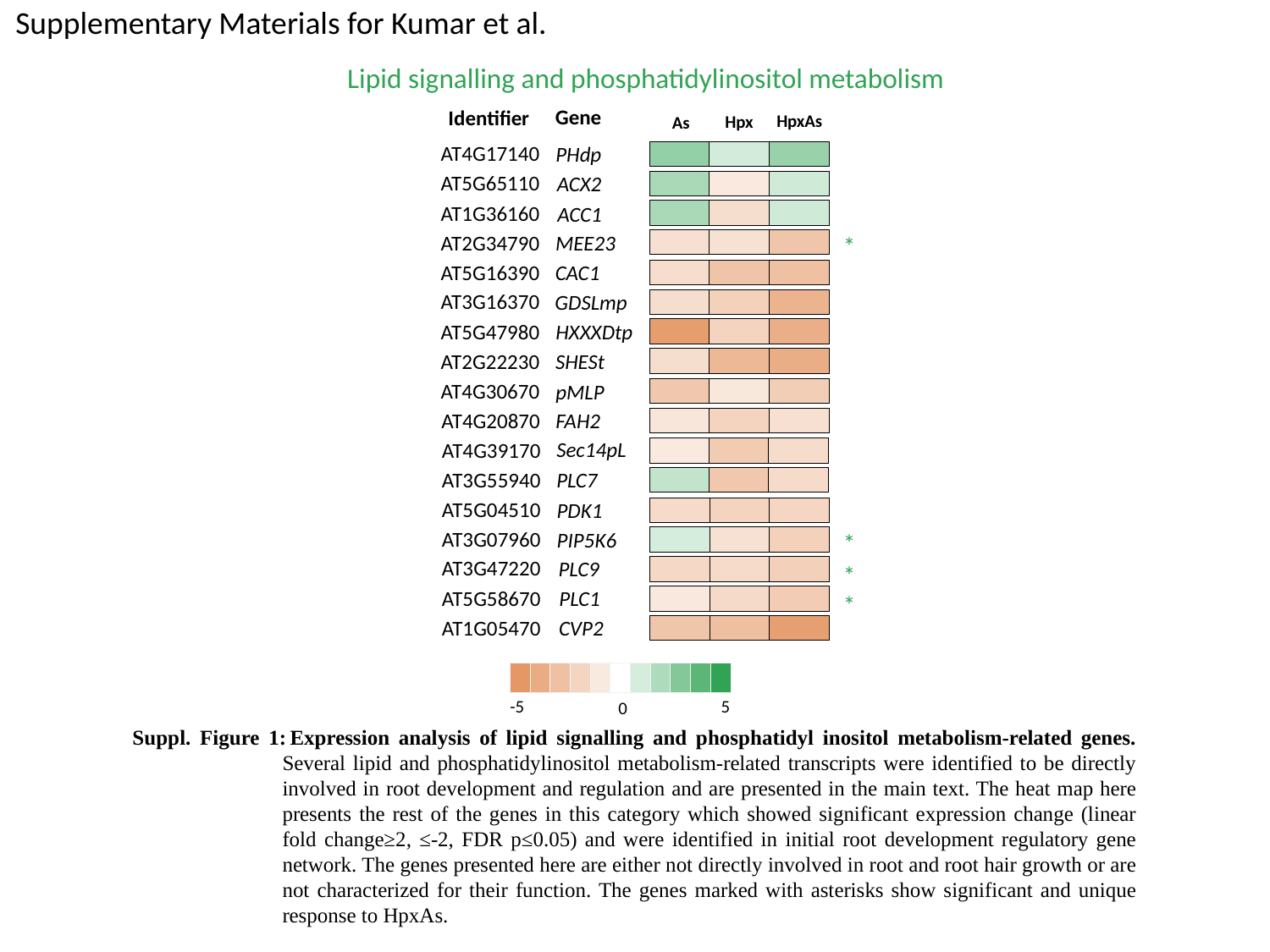

Supplementary Materials for Kumar et al.
Lipid signalling and phosphatidylinositol metabolism
Gene
PHdp
ACX2
ACC1
MEE23
CAC1
GDSLmp
HXXXDtp
SHESt
pMLP
FAH2
Sec14pL
PLC7
PDK1
PIP5K6
PLC9
PLC1
CVP2
Identifier
 AT4G17140
 AT5G65110
 AT1G36160
 AT2G34790
 AT5G16390
 AT3G16370
 AT5G47980
 AT2G22230
 AT4G30670
AT4G20870
 AT4G39170
 AT3G55940
 AT5G04510
 AT3G07960
 AT3G47220
 AT5G58670
 AT1G05470
HpxAs
Hpx
As
*
*
*
*
5
-5
0
Suppl. Figure 1:	Expression analysis of lipid signalling and phosphatidyl inositol metabolism-related genes. Several lipid and phosphatidylinositol metabolism-related transcripts were identified to be directly involved in root development and regulation and are presented in the main text. The heat map here presents the rest of the genes in this category which showed significant expression change (linear fold change≥2, ≤-2, FDR p≤0.05) and were identified in initial root development regulatory gene network. The genes presented here are either not directly involved in root and root hair growth or are not characterized for their function. The genes marked with asterisks show significant and unique response to HpxAs.

## Slide 2
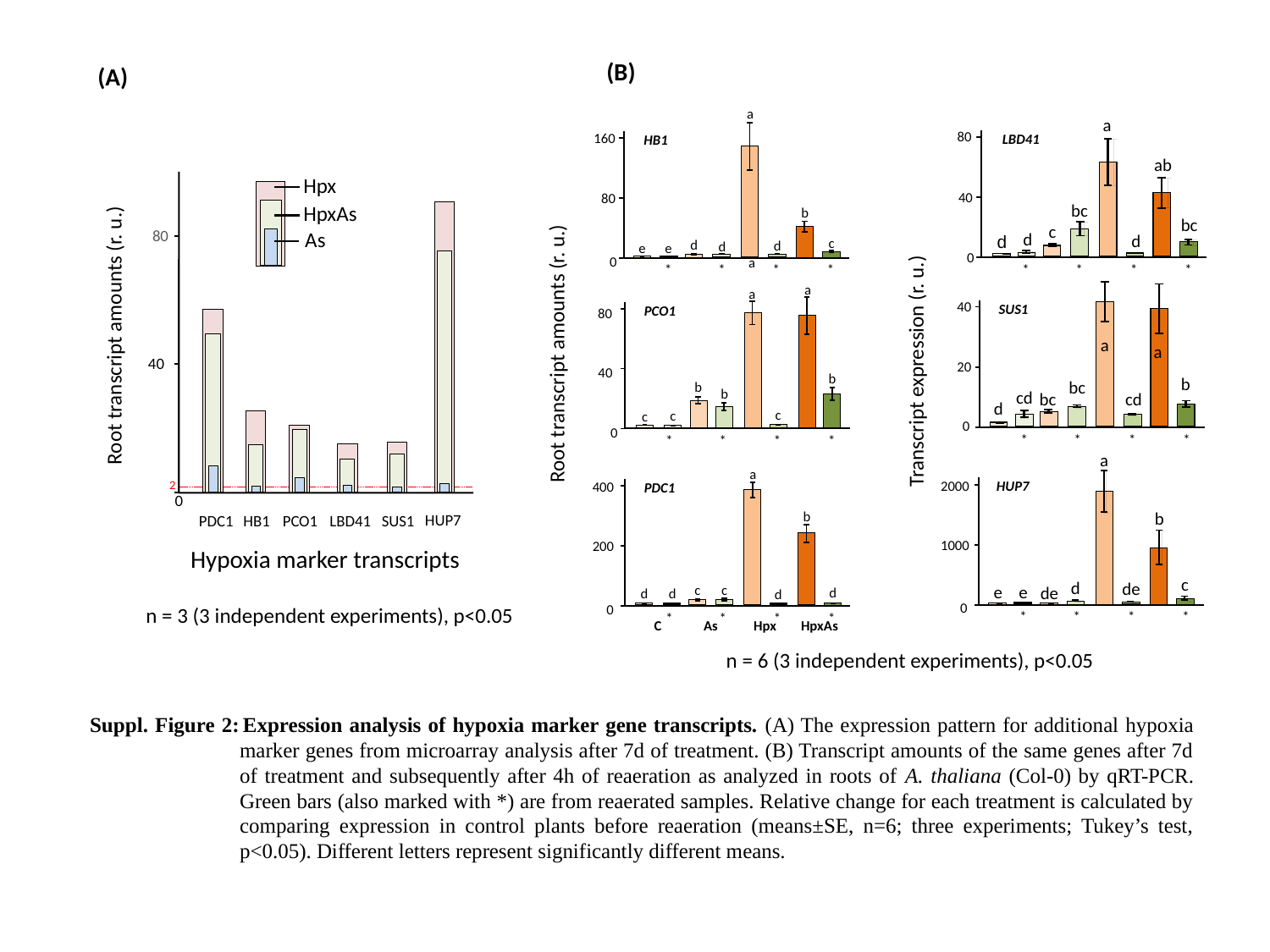

(B)
(A)
a
80
LBD41
ab
40
bc
bc
c
d
d
d
0
*
*
*
*
40
SUS1
20
b
bc
cd
bc
cd
d
0
*
*
*
*
a
a
Transcript expression (r. u.)
a
2000
HUP7
b
1000
c
d
de
e
e
de
0
*
*
*
*
a
160
HB1
80
b
c
d
d
d
e
e
0
a
*
*
*
*
a
a
PCO1
80
40
b
b
b
c
c
c
0
*
*
*
*
a
400
PDC1
b
200
c
c
d
d
d
d
0
*
*
*
*
As
Hpx
HpxAs
C
Root transcript amounts (r. u.)
n = 6 (3 independent experiments), p<0.05
Hpx
HpxAs
As
80
40
0
2
HUP7
PDC1
HB1
PCO1
LBD41
SUS1
Hypoxia marker transcripts
n = 3 (3 independent experiments), p<0.05
Root transcript amounts (r. u.)
Suppl. Figure 2:	Expression analysis of hypoxia marker gene transcripts. (A) The expression pattern for additional hypoxia marker genes from microarray analysis after 7d of treatment. (B) Transcript amounts of the same genes after 7d of treatment and subsequently after 4h of reaeration as analyzed in roots of A. thaliana (Col-0) by qRT-PCR. Green bars (also marked with *) are from reaerated samples. Relative change for each treatment is calculated by comparing expression in control plants before reaeration (means±SE, n=6; three experiments; Tukey’s test, p<0.05). Different letters represent significantly different means.

## Slide 3
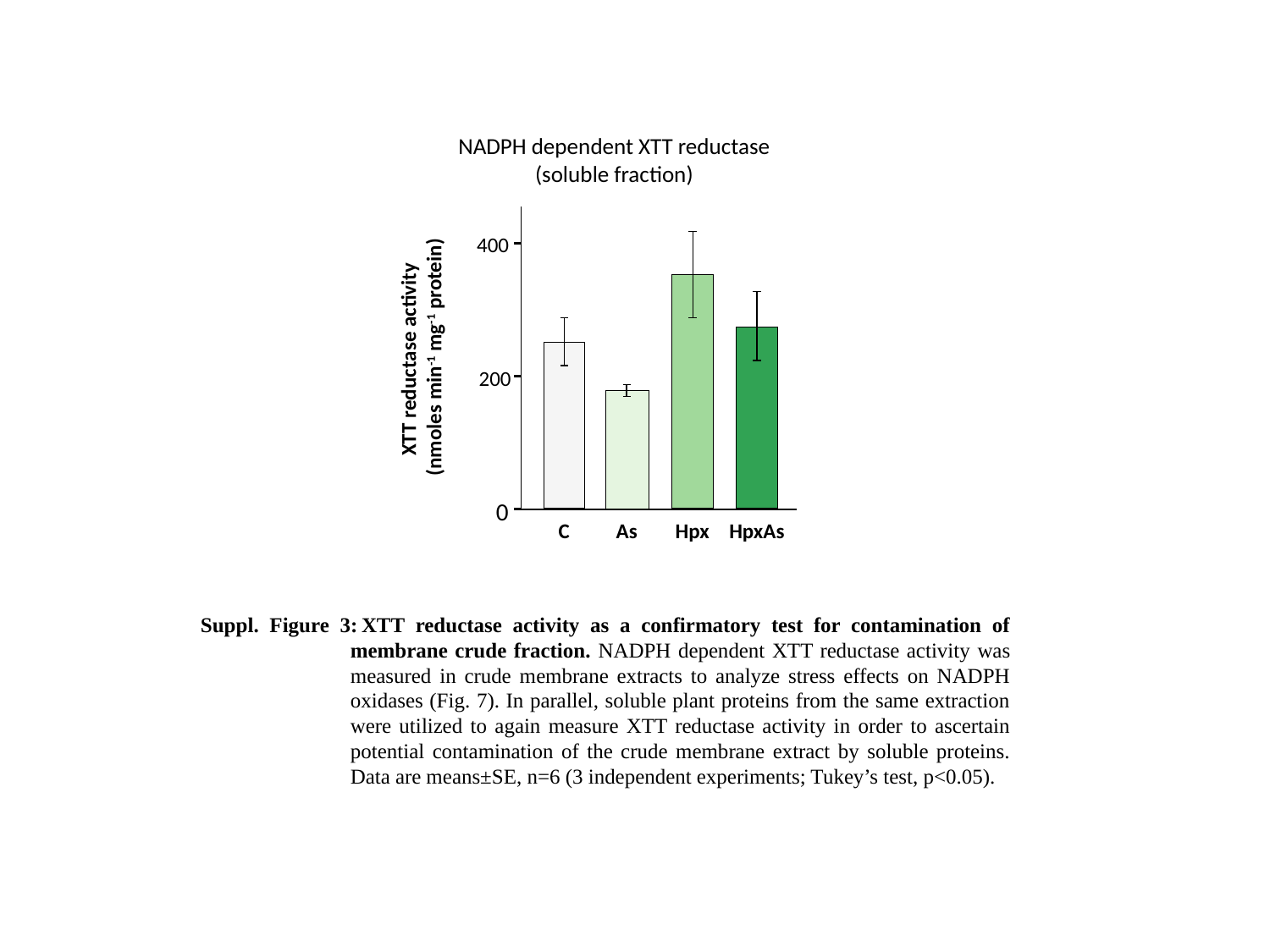

NADPH dependent XTT reductase
(soluble fraction)
400
XTT reductase activity
(nmoles min-1 mg-1 protein)
200
0
Hpx
C
As
HpxAs
Suppl. Figure 3:	XTT reductase activity as a confirmatory test for contamination of membrane crude fraction. NADPH dependent XTT reductase activity was measured in crude membrane extracts to analyze stress effects on NADPH oxidases (Fig. 7). In parallel, soluble plant proteins from the same extraction were utilized to again measure XTT reductase activity in order to ascertain potential contamination of the crude membrane extract by soluble proteins. Data are means±SE, n=6 (3 independent experiments; Tukey’s test, p<0.05).

## Slide 4
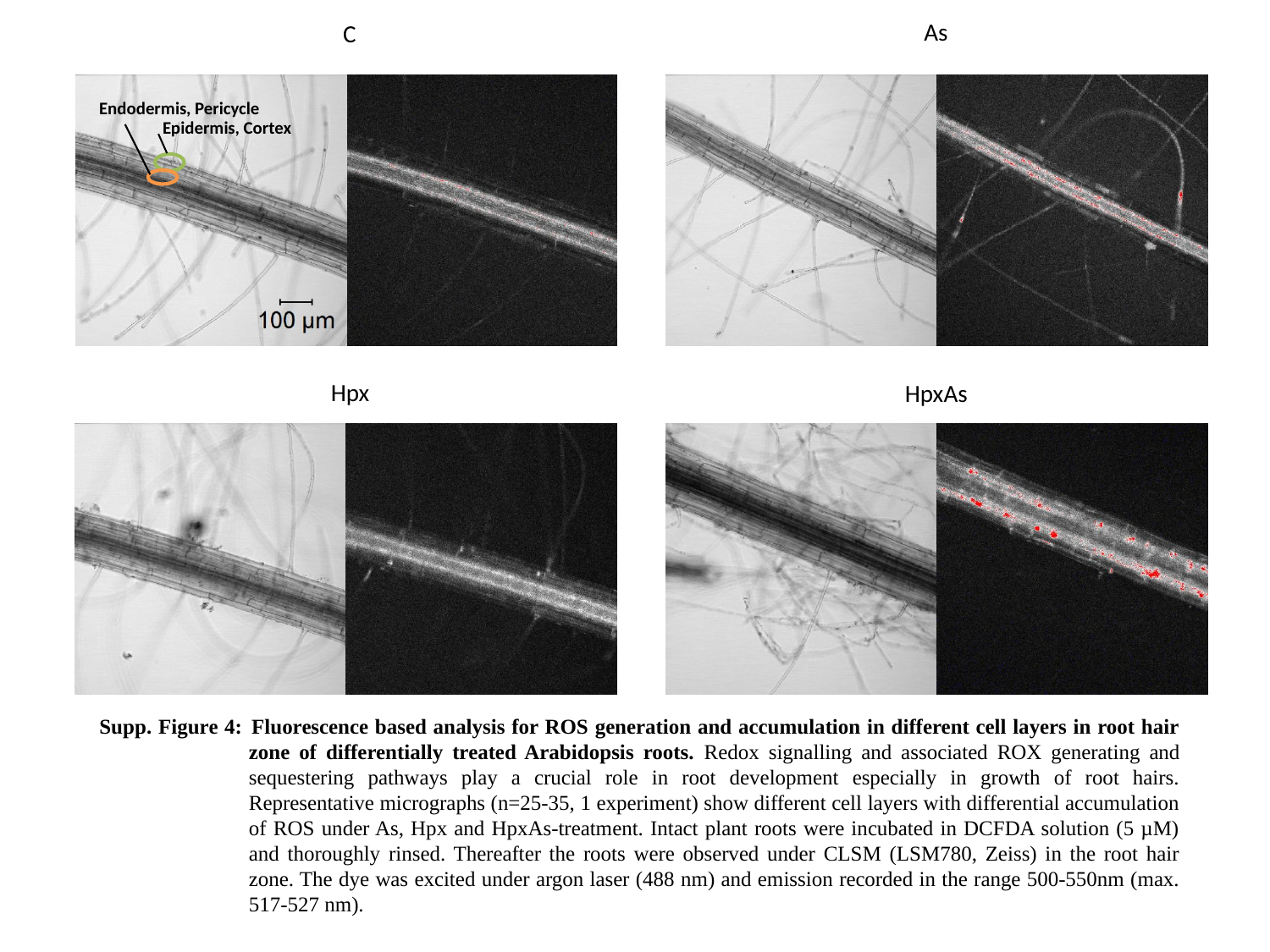

As
C
Hpx
HpxAs
Endodermis, Pericycle
Epidermis, Cortex
Supp. Figure 4:	Fluorescence based analysis for ROS generation and accumulation in different cell layers in root hair zone of differentially treated Arabidopsis roots. Redox signalling and associated ROX generating and sequestering pathways play a crucial role in root development especially in growth of root hairs. Representative micrographs (n=25-35, 1 experiment) show different cell layers with differential accumulation of ROS under As, Hpx and HpxAs-treatment. Intact plant roots were incubated in DCFDA solution (5 µM) and thoroughly rinsed. Thereafter the roots were observed under CLSM (LSM780, Zeiss) in the root hair zone. The dye was excited under argon laser (488 nm) and emission recorded in the range 500-550nm (max. 517-527 nm).

## Slide 5
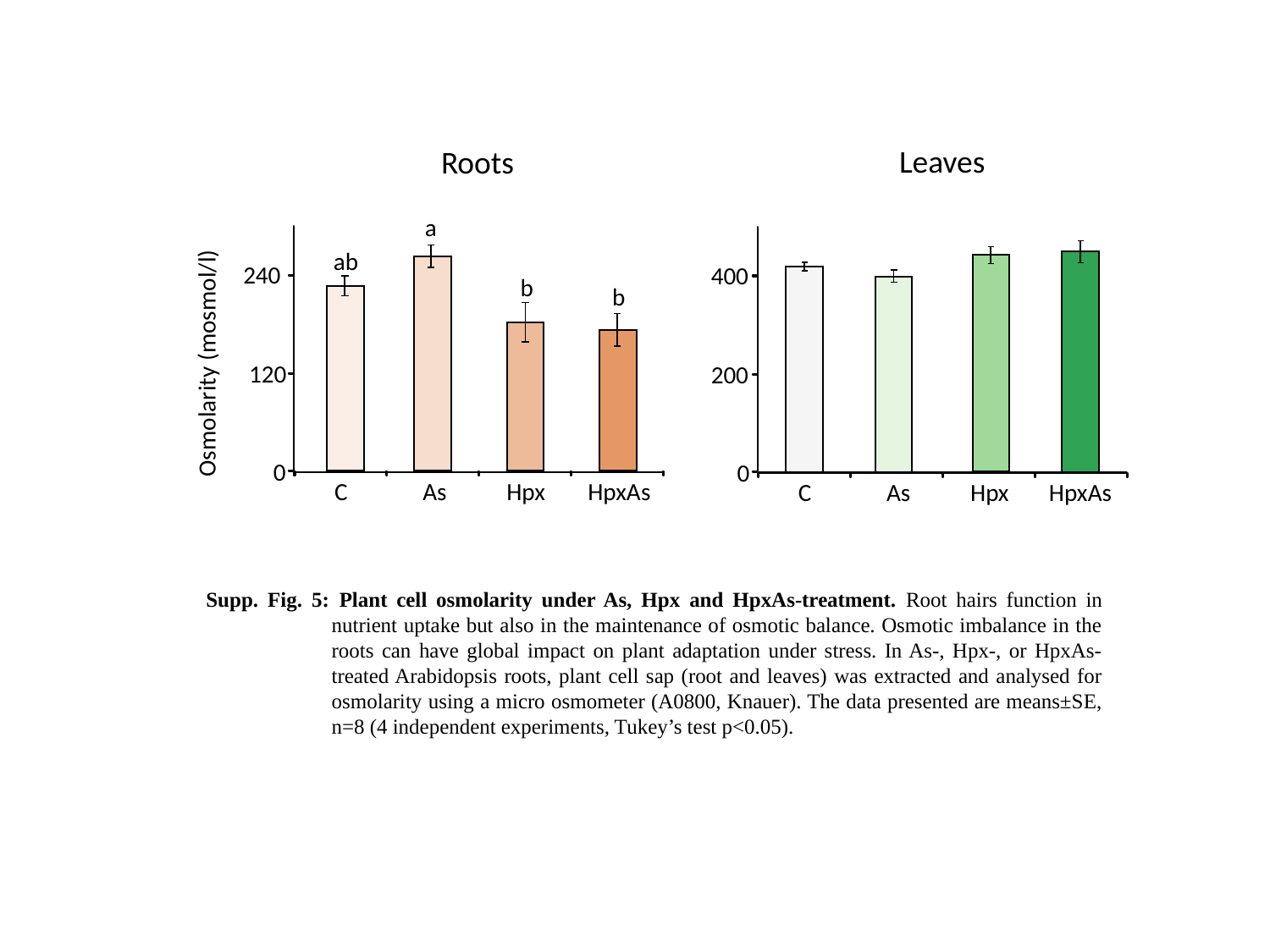

Leaves
400
200
0
C
As
Hpx
HpxAs
Roots
240
120
0
C
As
Hpx
HpxAs
Osmolarity (mosmol/l)
a
ab
b
b
Supp. Fig. 5:	Plant cell osmolarity under As, Hpx and HpxAs-treatment. Root hairs function in nutrient uptake but also in the maintenance of osmotic balance. Osmotic imbalance in the roots can have global impact on plant adaptation under stress. In As-, Hpx-, or HpxAs-treated Arabidopsis roots, plant cell sap (root and leaves) was extracted and analysed for osmolarity using a micro osmometer (A0800, Knauer). The data presented are means±SE, n=8 (4 independent experiments, Tukey’s test p<0.05).
